# Green Biosynthesis of Silver Nanoparticles Using *Annona glabra* and *Annona squamosa* Extracts with Antimicrobial, Anticancer, Apoptosis Potentials, Assisted by In Silico Modeling, and Metabolic Profiling

**DOI:** 10.3390/ph15111354

**Published:** 2022-11-02

**Authors:** Fatma A. Mokhtar, Nabil M. Selim, Seham S. Elhawary, Soha R. Abd El Hadi, Mona H. Hetta, Marzough A. Albalawi, Ali A. Shati, Mohammad Y. Alfaifi, Serag Eldin I. Elbehairi, Lamiaa I. Fahmy, Rana M. Ibrahim

**Affiliations:** 1 Department of Pharmacognosy, Faculty of Pharmacy, Al Salam University, Kafr Alzayat, Algharbia 31611, Egypt; 2 Pharmacognosy Department, Faculty of Pharmacy, Cairo University, Kasr-El-Ainy Street, Cairo 11562, Egypt; 3 Department of Pharmaceutical Chemistry, Faculty of Pharmacy, Egyptian Russian University, Cairo 11562, Egypt; 4 Department of Pharmacognosy, Faculty of Pharmacy, Fayoum University, Fayoum 63514, Egypt; 5 Department of Chemistry, Alwajh College, University of Tabuk, Tabuk 71491, Saudi Arabia; 6 Biology Department, Faculty of Science, King Khalid University, Alfra’a 62223, Saudi Arabia; 7 Cell Culture Lab, Egyptian Organization for Biological Products and Vaccines (VACSERA Holding Company), Giza 12654, Egypt; 8 Department of Microbiology and Immunology, Faculty of Pharmacy, October University for Modern Sciences and Arts (MSA), Giza 12573, Egypt

**Keywords:** *Annona*, UPLC-QTOF-MS/MS, silver nanoparticles, anticancer, apoptosis, antibacterial, antifungal, docking

## Abstract

*Annona glabra* L. (AngTE) and *Annona squamosa* L. (AnsTE) fruits have been widely used in cancer treatment. Accordingly, their extracts were used to synthesize silver nanoparticles via a biogenic route (Ang-AgNPs) and (Ans-AgNPs), respectively. Chemical profiling was established using UPLC-QTOF-MS/MS. All species were tested for anticancer activity against human cervical cancer cells (HeLa), prostate adenocarcinoma metastatic (PC3), and ovary adenocarcinoma (SKOV3) using sulphorhodamine B assay. Apoptosis was determined using Annexin flow cytometry along with cell cycle analysis and supported by a molecular docking. The antibacterial and synergistic effect when combined with gentamicin were evaluated. A total of 114 compounds were tentatively identified, mainly acetogenins and *ent*-kaurane diterpenes. AnsTE and Ans-AgNPs had the most potent cytotoxicity on HeLa and SKOV3 cells, inducing a significant apoptotic effect against all tumor cells. The AnsTE and Ans-AgNPs significantly arrested PC3, SKOV3, and HeLa cells in the S phase. The nanoparticles demonstrated greater antibacterial and antifungal activities, as well as a synergistic effect with gentamicin against *P. aeruginosa* and *E. coli*. Finally, a molecular docking was attempted to investigate the binding mode of the identified compounds in Bcl-2 proteins’ receptor, implying that the fruits and their nanoparticles are excellent candidates for treating skin infections in patients with ovarian or prostatic cancer.

## 1. Introduction

*Annona squamosa* L. and *A. glabra* L., two species in the family Annonaceae, have been widely investigated for their therapeutic potential, considering that the major components of Annonaceous plants are acetogenins. Both species contain various phytochemicals including alkaloids, flavonoid glycosides, phenolics, cyclopeptides, and volatile oils. Recently, extracts of *A. squamosa* and *A. glabra* have shown antiproliferative effects against several cancer types such as lung, colon, and breast cancer cells by inhibiting mitochondrial complex I, which is related to oxidative phosphorylation and ATP synthesis [1,2]. Acetogenins trigger cell apoptosis by disrupting mitochondrial membrane potential in cancer cells. Collectively, these results suggest that the phytochemical constituents such as acetogenins and diterpenes might contribute substantially to the antineoplastic effects of *A. squamosa* and *A. glabra* against cancer cell lines [3,4]. Furthermore, to evaluate the potential of these phytochemical constituents for the treatment of cancer, we tested molecular docking analyses against antiapoptotic proteins.

Liquid chromatography coupled with high-resolution mass spectrometry (LC/HR-MS) could be a promising tool in metabolic profiling because it can detect a wide range of metabolites simultaneously and at a high throughput [5]. Many researchers have recently used LC/MS to identify and characterize various bioactive compounds [6]. It yields elemental composition and accurate mass data (0.5–5 ppm) for each compound. The MS/MS fragmentation obtained with a Q-TOF or LTQ-Orbitrap could be used to accurately determine the chemical structures of the compounds [7]. In the absence of commercial standards, this step is extremely useful [5].

A great method for producing uniform biogenic, environmentally friendly, and inexpensive nanoparticles with good stability and low toxicity is the formation of silver nanoparticles using a plant extract as a reducing and capping agent [8]. The biogenic nanoparticles enhance the biological activities to some extent more than the chemical, physical or radiation-induced nanoparticles and with lower toxicity [9].

Research in possible cancer treatment protocols and alternatives to inhibit cancer progression and its underlaying mechanisms is developing at an increasing rate. These studies aim to control the deadly progression of cancer cells in an effective, rapid, safe, and non-toxic manner, that can decrease or overcome chemotherapy and radiation treatments’ side effects [10,11].

Anticancer activities of extracted and derived green synthesized nanoparticles have recently gained great importance. They are considered a basic line in anticancer development research and anticipated in documenting the possible contribution of the plant kingdom in anticancer medication development and natural molecular biology studies [12,13]. However, no comparative studies on the chemical characterization or anticancer potential of the two fruits or their green nanoparticles were discovered.

Resistant microorganisms are increasing rapidly on our planet and could be considered a public health top threat. It manifested itself as a reaction to the progressive abuse of chemical agents, especially unauthorized disinfectants and sterilizing agents [14]. Therefore, a return to natural antimicrobial agents from plant origin is the solution, hence natural antimicrobials alone or in combination with standard antibiotic is one of the top priorities in our scientific research to develop safe and effective antimicrobial agents [15].

This extensive study’s objective was to compare the metabolic profiles of *A. squamosa* and *A. glabra* fruits using UPLC-HR-QTOF/MS/MS, for the first time, a correlation of metabolites profiles with cytotoxic and antimicrobial activities was performed. Additionally, the two fruits’ greenly synthesized silver nanoparticles and their influence on the cytotoxic activities were for the first time characterized and assessed.

## 2. Results

### 2.1. UPLC-QTOF-MS/MS Metabolic Profiling of the Fruits

The chemical profiles of ethanolic extracts of *A. squamosa* and *A. glabra* have been analyzed using UPLC-HR-QTOF/MS/MS to highlight the potential active constituents in the extracts. In total, 114 compounds have been identified in the positive ionization mode, including 77 acetogenins, 20 diterpenes, and 17 miscellaneous compounds. The detected compounds were identified using the dictionary of natural products (2015) and were compared with the available literature [16,17,18,19,20,21]. The molecular ions, retention times (min), molecular formulas, major fragment ions, and the identities of the identified compounds are recorded in Table S1. The UPLC chromatograms of *A. squamosa* and *A. glabra* ethanolic extracts are shown in Figure S1. 

#### 2.1.1. Acetogenins

Natural substances known as annonaceous acetogenins (ACGs) are only present in the Annonaceae species. They are fatty acid derivatives that have an alkanol moiety attached to the 2,4-disubstituted -lactone ring at position 2, where a long hydrocarbon chain that frequently contains a number of oxygenated moieties such as hydroxyl, acetoxy, carbonyl, and/or a double bond typically contains variable numbers (1–4) of tetrahydrofuran rings (THF). The ACGs can also be divided into subgroups based on how many and how the THF rings are arranged within each molecule. These subgroups include mono-THF, adjacent bis-THF, nonadjacent bis-THF, nonadjacent THF or non-THF, and adjacent tris-THF. Based on the accurate mass measurements, fragmentation patterns, and available literature, 77 different ACGs were tentatively identified in the extracts of *A. squamosa* and *A. glabra* using UPLC-QTOF technique. 

According to the amount of THFs, the detected ACGs were divided into four subclasses: adjacent bis-THF, non-adjacent bis-THF, mono-THF with one adjacent-OH group, and mono THF with OH groups on both sides. In their mass spectra, ACGs are mainly identified as sodium adducts [M+Na]^+^. The removal of the lactone moieties results in the ACGs’ MS^2^ spectra exhibiting distinctive fragment ions (−112 amu). The hydrocarbon chain’s length, the positions of the hydroxyl groups, and the positions of the THF rings may all be calculated thanks to the fragmentation around the hydroxyl groups. The amount of hydroxyl groups contained in the molecule is shown by the subsequent loss of water (−18 amu).

Squadiolin C is one of the mono-THFs with an adjacent OH group that can serve as a model. It contained a protonated molecular ion with the formula C_35_H_65_O_7_^+^ at *m*/*z* 597.4731 (peak 61, Table S1). The fragmentation behavior of Squadiolin C showed characteristic ions due to the successive loss of four water molecules from the parent ion. This was obvious from the ions formed at *m*/*z* 579, 561, 543, and 525, indicating the presence of four OH groups in its structure. The remaining acetogenins (Table S1) were identified following the same dissociation pathways and were compared with the literature [18,22,23].

The mono-THF with two adjacent hydroxyl groups were also identified in the ethanolic extracts of the two fruits. Annonacinone (peak 79, Table S1) is an example. It exhibited a molecular ion at *m*/*z* 617.4393 [M+Na]^+^, corresponding to the molecular formula C_35_H_62_O_7_. Its mass spectra revealed fragment ions at *m*/*z* 577 [(M+H)^+^–H_2_O]^+^, 559 [(M+H)^+^–2H_2_O]^+^, and 541 [M+H–3H_2_O]^+^, which suggests the existence of three OH groups, caused by the sequential loss of three water groups.

Bullatacin is an example of the nearby bis-THF ACGS that have been found (peak 87, Table S1). It had a molecular ion with the chemical formula C_37_H_67_O_7_^+^ at *m*/*z* 623.4863. The Na adduct is what causes the peak at *m*/*z* 645.4683. The consecutive losses of water molecules from the parent ion produced the mass fragments at *m*/*z* 587 [(M+H)^+^–2H_2_O]^+^ and 569 [(M+H)^+^–3H_2_O]^+^, indicating the existence of three hydroxyl groups.

Squamostatin B (peak 30, Table S1) was one of the discovered non-adjacent bis-THF ACGS. It displayed a sodium adduct peak at *m*/*z* 661.4632 [M+Na]^+^, which is consistent with the molecular formula C_37_H_67_O_8_^+^, and a molecular ion at *m*/*z* 639.4812 [M+H]^+^. Its Ms^2^ spectrum revealed the four hydroxyl groups’ distinctive fragments at *m*/*z* 621 [(M+H)^+^–H_2_O]^+^, 603 [(M+H)^+^–2H_2_O]^+^, 585 [(M+H)^+^–3H_2_O]^+^, and 567 [(M+H)^+^–4H_2_O]^+^, which confirms the presence of four hydroxyl groups.

#### 2.1.2. Diterpenes

Diterpenoids called ent-kauranes are typically found in Annonaceous plants and play a supporting role in the production of gibberellin plant growth hormones. They exhibit a variety of biological properties, including anti-inflammatory, antibacterial, and anticancer properties. Based on their MS/MS fragmentation and published literature, twenty ent-kauranes were inferred to be present in the ethanolic extracts. Their MS/MS spectra were dominated by the diagnostic fragment ions created by the loss of water and carbon monoxide molecules.

A molecular ion at *m*/*z* 363.2499 (C_22_H_35_O_4_^+^) and a base peak at *m*/*z* 289 due to the loss of the (CH_2_OCOCH_3_)^+^ ion were both present in Annoglabasin B (compound 70, Table S1). At *m*/*z* 271 and 234, respectively, where a water molecule and subsequently a CO group were successively lost, other distinguishing ions were also visible. Annoglabasin D (compound 17, Table S1) had a protonated molecular ion at *m*/*z* 391.2489 corresponding to the formula C_23_H_35_O_5_^+^. In its mass spectrum it gives the fragment ions at *m*/*z* 331, 300, and 272 due to successive loss of acetoxy, methoxy, and CO groups, respectively.

Annoglabasin E (compound 48 in Table S1) included a protonated molecular ion with the chemical formula C_20_H_33_O_3_^+^ at *m*/*z* 321.2398. The molecular ion’s removal of the CH_2_OH group is indicated by the base peak at *m*/*z* 289 that was recorded. At *m*/*z* 271 and 243, respectively, further fragment ions were discovered because of the loss of an H_2_O and a CO group.

Annoglabasin F (compound 21, Table S1) showed a molecular ion at *m*/*z* 379.2423 corresponding to the formula C_22_H_35_O_5_^+^ and a base peak at *m*/*z* 361 due to the loss of hydroxyl group. Additionally, the peaks that appeared at *m*/*z* 302, 271, and 243 were formed from the successive loss of COOCH_3_, OCH_3_, and CO groups, respectively. Following the same fragmentation pathway, the remaining ent-kauranes were assigned and their identities were recorded in Table S1.

### 2.2. Characterization of Silver Nanoparticles

#### 2.2.1. UV Spectroscopy

**Figure 1:**
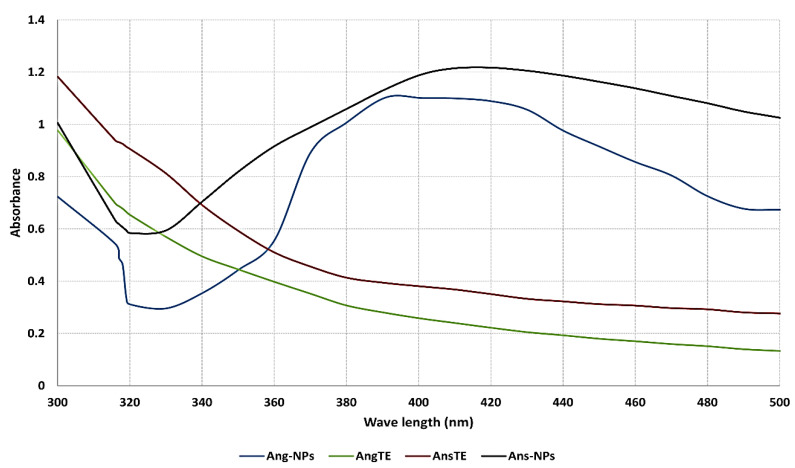
UV spectrum of the biosynthesized silver nanoparticles Ang-AgNPs and Ans-AgNPs compared to their extracts AngTE and AnsTE, respectively.

UV-vis spectroscopy is the first analysis parameter used as an indicator for the production of stable silver nanoparticles. Due to nanoparticles’ selective UV absorbance, it is thought to be the quickest and easiest way to achieve accurate evaluation for nanoparticle synthesis. AgNPs exhibit a distinctive optical reflectivity that causes them to interact strongly with certain light wavelengths. Their collective electron oscillation results in a surface Plasmon resonance (SPR) absorption band that is produced by free electrons. AgNP absorption is influenced by the dielectric medium as well as chemical surroundings, particle shape, and particle size. The produced Ang-AgNPs and Ans-AgNPs were subjected to UV measurements, which revealed absorbance at 392 nm and 414 nm, respectively (Figure 1). 

#### 2.2.2. FTIR

**Figure 2:**
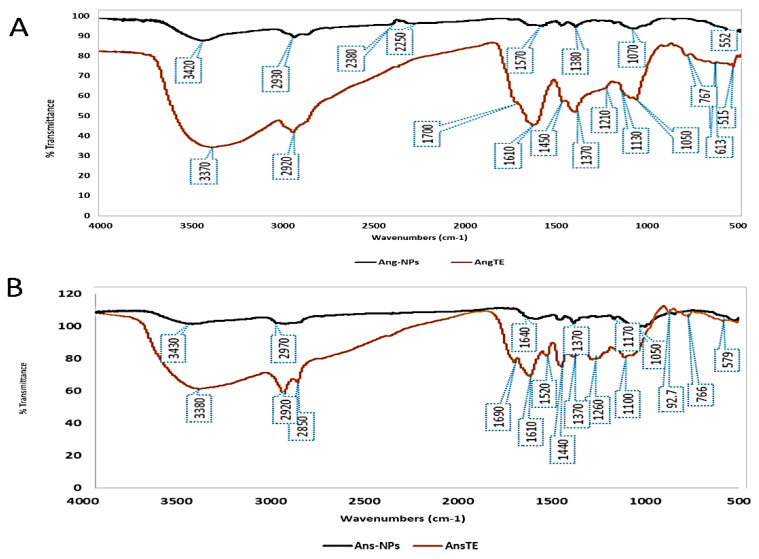
FTIR spectrum of the biosynthesized silver nanoparticles, (**A**): Ang-AgNPs and (**B**): Ans-AgNPs compared to their respective extracts, AngTE and AnsTE.

By using FTIR spectroscopy measurements, it was possible to estimate which functional chemical groups of the AngTE and AnsTE oversaw the reduction reaction that contributed to the creation of the silver nanoparticles. Peaks at 3480, 2920, and 1610 cm^−1^ indicate the functional groups in AnsTE, which are OH, C aliphatic, and C=O of phenolic acids and flavonoids. Peaks at 3470, 2920, and 1610 cm^−1^ represent the functional groups in AngTE. While the peaks at 1450 cm^−1^ and 1440 cm^−1^ in AngTE and AnsTE, respectively, demonstrated the presence of polyphenols and aromatic compounds, the peaks at 1050 cm^−1^ and 1100 cm^−1^ in AngTE and AnsTE, respectively, confirmed the presence of secondary OH groups (Figure 2).

#### 2.2.3. High-Resolution Transmission Electron Microscope (HR-TEM)

**Figure 3:**
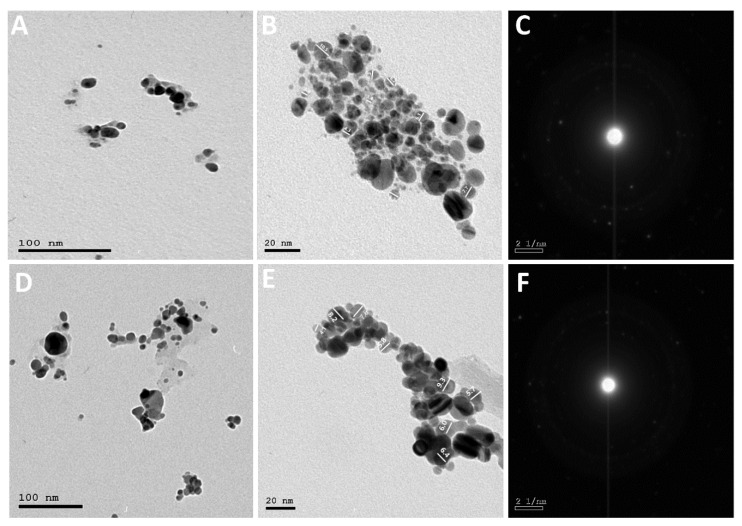
HR-TEM of silver nanoparticles biosynthesized using the extracts, AngTE and AnsTE; (**A**,**D**) at 100 nm, (**B**,**E**); at 20 nm, (**C**,**F**): SAED for Ang-AgNPs and Ans-AgNPs in the same order.

By employing *A. glabra* and *A. squamosa* extracts as a reducing agent, naturally occurring silver nanoparticles were produced. The spherical shape of the AgNPs was confirmed by transmission electron microscopy. In the same sequence, the two Annona species produce AgNPs with average particle sizes of 7.11 and 6.63 nm for Ang-AgNPs and Ans-AgNPs [8] (Figure 3). Selected Area Electron Diffraction Pattern (SAED) evidenced the crystalline nature of both Ang-AgNPs and Ans-AgNPs.

#### 2.2.4. Zeta Potential and DLS

**Figure 4:**
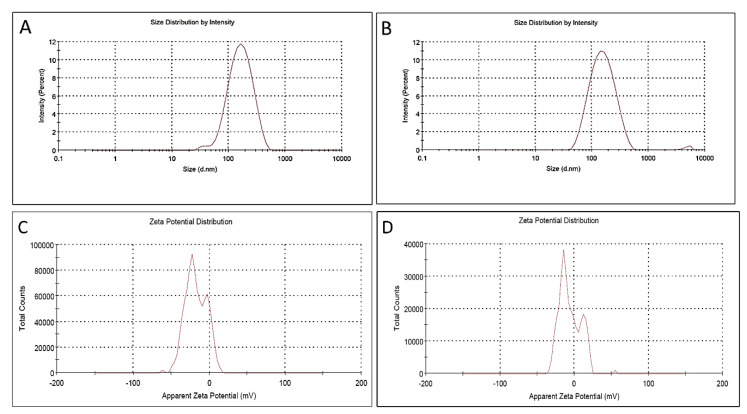
Zeta potential measurements of the biosynthesized silver nanoparticles using the extracts, AngTE and AnsTE; (**A**,**B**): particles size distribution of Ang-AgNPs and Ans-AgNPs, respectively, (**C**,**D**): particles charges of Ang-AgNPs and Ans-AgNPs, respectively.

Nanoparticles stability, nanocharge, and surrounding media size were indicated to the Ang-AgNPs and Ans-AgNPs by Zeta potential technique. The application of the zeta potential technique was employed to evaluate the surface nano charges. The zeta potential gave a value of −17.7.0 ± 0.7 mV and −6.0 ± 0.09 mV to the nanoparticles Ang-AgNPs and Ans-AgNPs, respectively, formed through *A. glabra* and *A. squamosa*. The stabilities of the formed nanosilver particles are indicated by the negative charges of the Ang-AgNPs and Ans-AgNPs (Figure 4).

#### 2.2.5. X-ray Diffraction (XRD)

**Figure 5:**
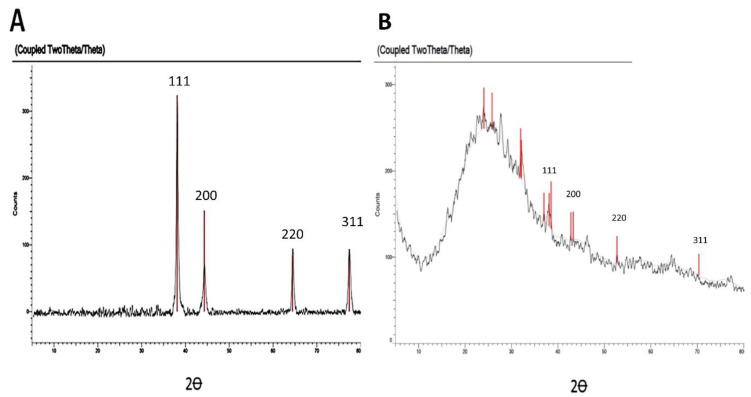
X-ray diffraction pattern of the biosynthesized AgNPs using (**A**): *A. glabra* and (**B**): *A. squamosa*.

Diffraction silver nano peaks obtained for the formed AgNPs by *A.glabra*, were represented by peaks on the 2 Ꝋ scale at 38.45°, 44.31°, 64.80°, and 77.81°, respectively ascribed to (111), (200), (220), and (311) planes (Figure 5), while silver nano peaks obtained for the formed AgNPs by *A. squamosa*, were represented by peaks on the 2 Ꝋ scale at 38.24°, 45.06°, 64.55°, and 77.73°, respectively ascribed to (111), (200), (220), and (311) planes (Figure 5). These results are evidence of the formation of pure silver nanoparticles in crystalline nature for both Ang-AgNPs and Ans-AgNPs. The findings are consistent with previous reports on synthesis of silver nanoparticles [24].

#### 2.2.6. Scanning Electron Microscope (SEM)

**Figure 6:**
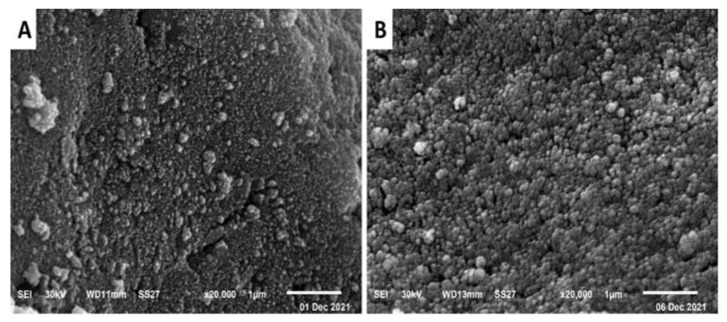
SEM of silver nanoparticles biosynthesized using (**A**): *A. glabra* and (**B**): *A. squamosa*, photographs at ×20,000.

SEM can precisely demonstrate the surface appearance of the biosynthesized silver nanoparticles, the particle size, shape, and distribution of the nanoparticles, in addition to determining of the micro or nanoscale formation. The SEM analysis of the biosynthesized silver nanoparticles from *A. glabra* and *A. squamosa* revealed the spherical nature and aggregate tendency of Ang-AgNPs and Ans-AgNPs (Figure 6).

### 2.3. The Biological Study

#### 2.3.1. Antimicrobial Evaluation of the Extracts and Their Biogenic Nanoparticles

**Figure 7:**
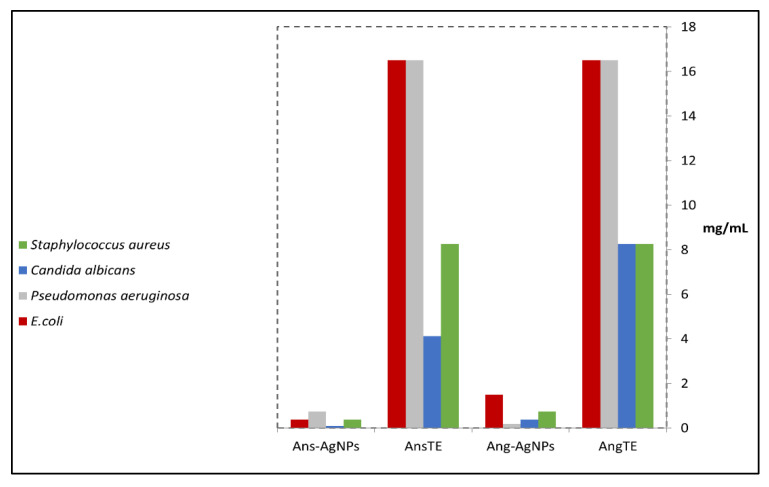
Results of the minimum inhibitory concentration (MIC) of the extracts, AngTE and AnsTE and their corresponding biogenic silver nanoparticles, Ans-AgNPs and Ang-AgNPs towards *P. aeruginosa*, *E. coli*, and *C. albicans*.

In the present study, the results show that the extracts possess antimicrobial activities against the tested microorganisms. The Ans-AgNPs and Ang-AgNPs showed more activity than that observed by AngTE and AnsTE activities. The MIC of AngTE and AnsTE was shown different values ranging from 16.5 until 8.25 mg/mL in (Figure 7), however Ans-AgNPs and Ang-AgNPs had MIC values varying between 0.75 mg/mL and 0.093 mg/mL. The results indicated that the Ans-AgNPs and Ang-AgNPs have stronger activity than that of AngTE and AnsTE. The Ans-AgNPs has the highest activity with a lower MIC value (0.093 mg/mL) against *C. albicans* while Ang-AgNPs has recorded the highest activity against *P. aeruginosa* with 0.18 mg/mL (Table S2).

The green synthesized nanoparticles were more active materials towards the *P. aeruginosa* and *E. coli*, so we went deeper to evaluate the synergistic potential of the nanoparticles with gentamicin and vancomycin by evaluating the zone of inhibition (Table 1, Table 2 and Table 3).

In a combination assay of Ans-AgNPs and Ang-AgNPs with gentamicin, they have shown a higher synergistic effect towards *P. aeruginosa* and *E. coli*, respectively. However, the assay of Ans-AgNPs with vancomycin did not show a higher synergistic effect towards S. aureus than standard antibiotic vancomycin. The difference in the synergistic effect between the different bacterial strains might be due to the thin peptidoglycan layer in case of Gram-negative strains, which facilitates the penetration of the nanoparticle extracts (Ans-AgNPs and Ang-AgNPs).

The synergistic effect towards *P. aeruginosa* and *E. coli* were proven through the measuring of the combination’s (formed silver nanoparticles+ gentamicin) inhibition zone and compared to the gentamicin and silver nanoparticles individually.

#### 2.3.2. Anticancer Activity

##### In Vitro Cytotoxic Activity

**Figure 8:**
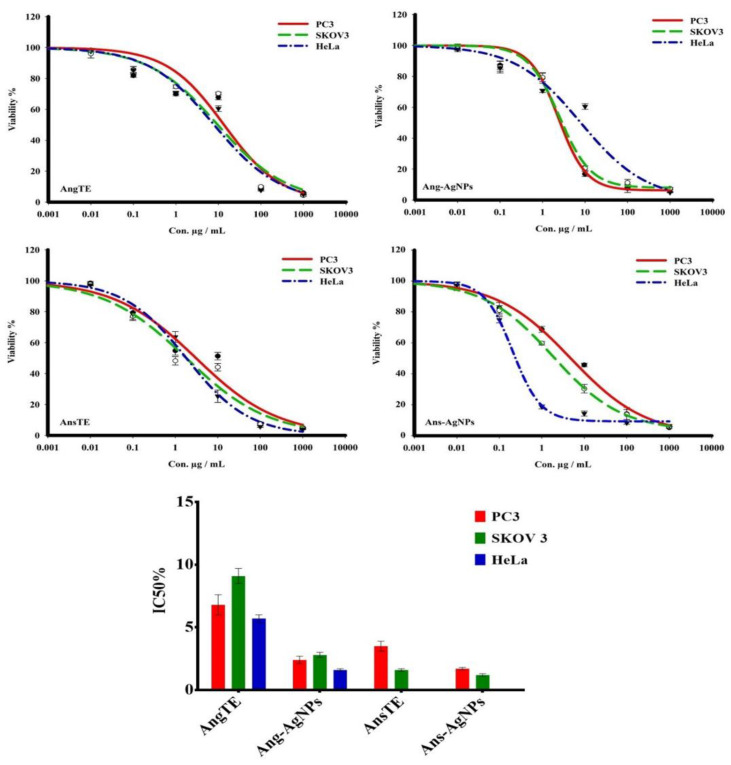
The toxicity response of the extracts, AngTE, AnsTE, and their nanoparticles, Ang-AgNPs, and Ans-AgNPs, respectively, to human cancer cells PC3, SKOV3, and HeLa. For 72 h, cells were incubated with variable concentrations of materials. SRB staining was used to determine cell viability.

The ethanolic extracts of *A. glabra* (AngTE), *A. squamosa* (AnsTE), and their nanoparticles, Ang-AgNPs and Ans-AgNPs, respectively, were tested using the SRB assay for their in vitro cytotoxic effects against the PC3, SKOV 3, and HeLa cancer cell lines at doses ranging from 0.01 to 1000 g. All samples tested positive for cytotoxicity against cancer cells in a variety of ways. The cytotoxicity of AnsTE and Ans-AgNPs on HeLa and SKOV 3 cells was the most potent, with IC_50_s ranging from 0.001 ± 0.0001 to 1.6 ± 0.1 g/mL, and the toxicity of Ans-AgNPs on PC3 cells, with IC_50_ of 1.7 ± 0.4. Additionally, the AngTE exerted a good potential killing effect against PC3, SKOV 3, and HeLa cancer cells, with IC_50_ of 6.8 ± 0.8, 9.1 ± 0.6, and 5.7 ± 0.3 µg/mL, respectively, while that of AnsTE on PC3 cell line was IC_50_ 3.5 ± 0.1 µg/mL. Moreover, Ang-AgNPs has the strongest cytotoxic effect on PC3 and SKOV 3 with IC_50_ 2.4 ± 0.3 and 2.8 ± 0.23 µg/mL, respectively (Figure 8 and Table S3).

**Figure 9:**
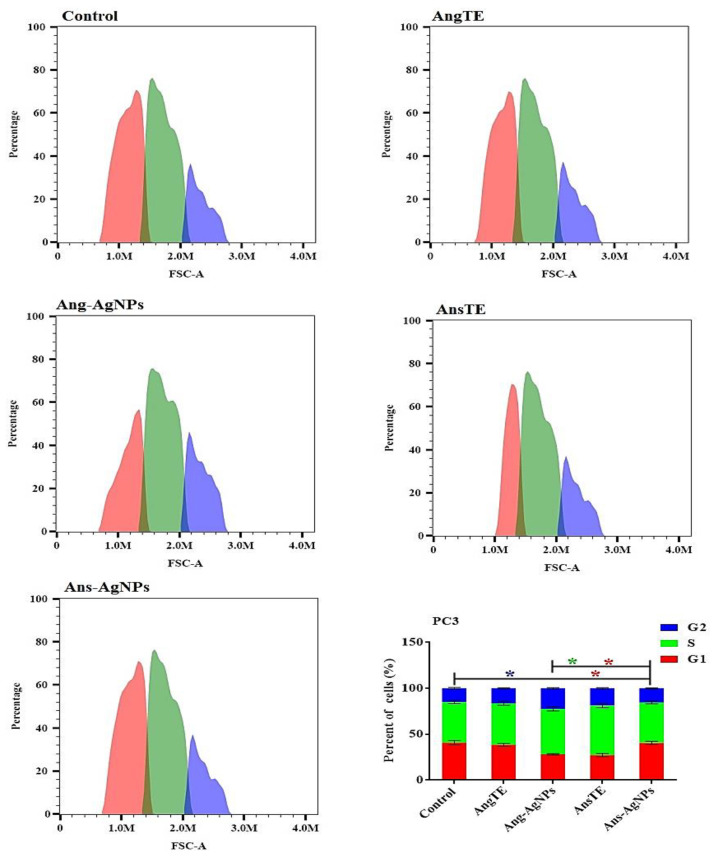
Effect of the extracts, AngTE, AnsTE, and their nanoparticles, Ang-AgNPs, and Ans-AgNPs, respectively, on cell cycle distributions of PC3 cells. Cell cycle distribution was determined using DNA cytometry analysis after exposure to AngTE, Ang-AgNPs, AnsTE, and Ans-AgNPs for 48 h. Data are presented as the mean ± SD; n = 3; G1, G2 and S are cell cycle phases, One-way ANOVA was used to test for statistical difference (* *p* < 0.05).

**Figure 10:**
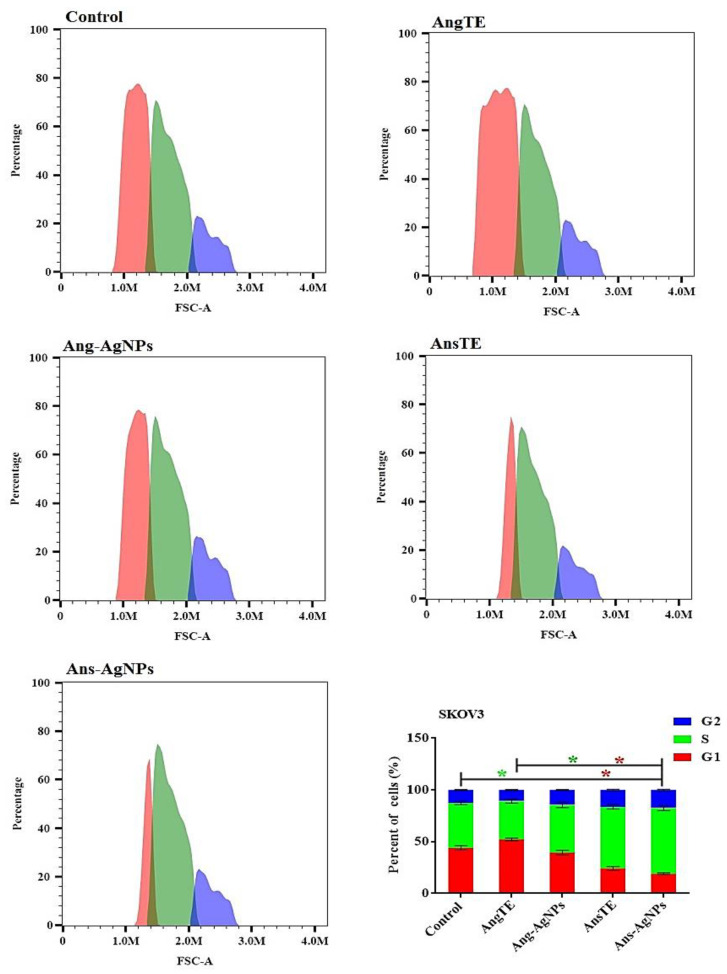
Effect of the extracts, AngTE, AnsTE, and their nanoparticles, Ang-AgNPs, and Ans-AgNPs, respectively, on cell cycle distributions of SKOV3 cells. Cell cycle distribution was determined using DNA cytometry analysis after exposure to AngTE, Ang-AgNPs, AnsTE, and Ans-AgNPs for 48 h. Data are presented as the mean ± SD; n = 3; G1, G2 and S are cell cycle phases, One-way ANOVA was used to test for statistical difference (* *p* < 0.05).

**Figure 11:**
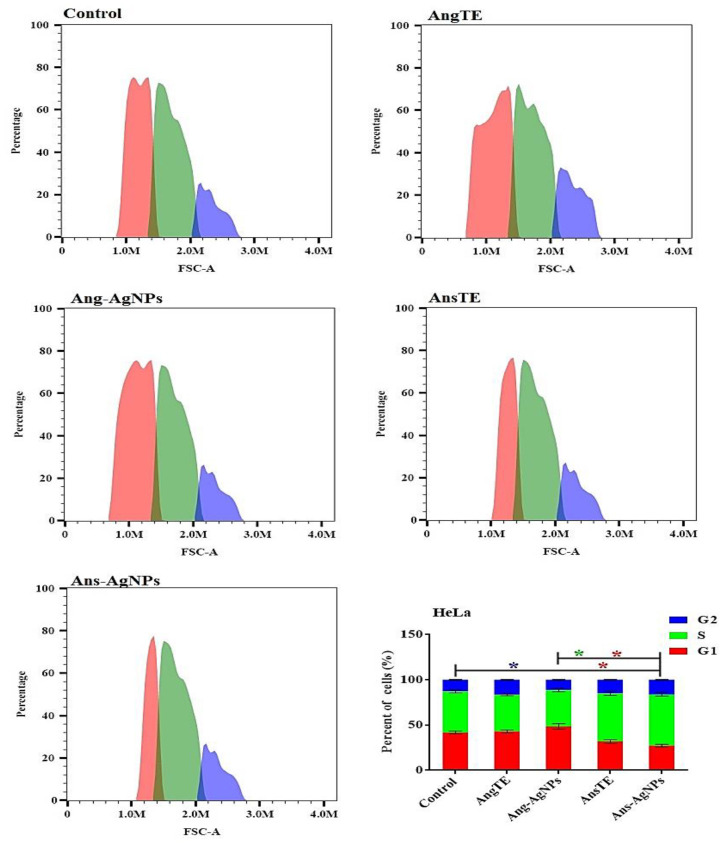
Effect of the extracts, AngTE, AnsTE, and their nanoparticles, Ang-AgNPs, and Ans-AgNPs, respectively, on cell cycle distributions of HeLa cells. Cell cycle distribution was determined using DNA cytometry analysis after exposure to AngTE, Ang-AgNPs, AnsTE, and Ans-AgNPs for 48 h. Data are presented as the mean ± SD; n = 3; G1, G2 and S are cell cycle phases, One-way ANOVA was used to test for statistical difference (* *p* < 0.05).

The Effect on the Cell Cycle Distribution of Solid Tumor Cells Tracking tumor cell cycle phases was used to investigate anticancer effects. As a result, the effect of AngTE, Ang-AgNPs, AnsTE, and Ans-AgNPs on the distribution of cell cycle phases in PC3, SKOV3, and HeLa cells after 48 h of treatment in cancer cells was studied using flow cytometry as shown in (Figure 9, Figure 10 and Figure 11), AnsTE and Ans-AgNPs significantly arrested PC3, SKOV3, and HeLa cells in the S phase with a ratio ranging from 44.1 ± 1.5% to 63.5 ± 2.3%, where the effect of Ang-AgNPs on stopping the PC3, SKOV3 cells in the S phase was 49.5 ± 2.2% and 46 ± 2.2%, respectively. In the meantime, after treatment with AngTE and Ang-AgNPs, the percentage of G1 phases in SKOV3 and HeLa cells increased by 42.8 ± 1.6% and 48.5 ± 3.1%, respectively, and treatment with AngTE affected the G1 phase by 38.7 ± 1.1% in SKOV3.

##### Cell Apoptosis Using Annexin V-FITC

**Figure 12:**
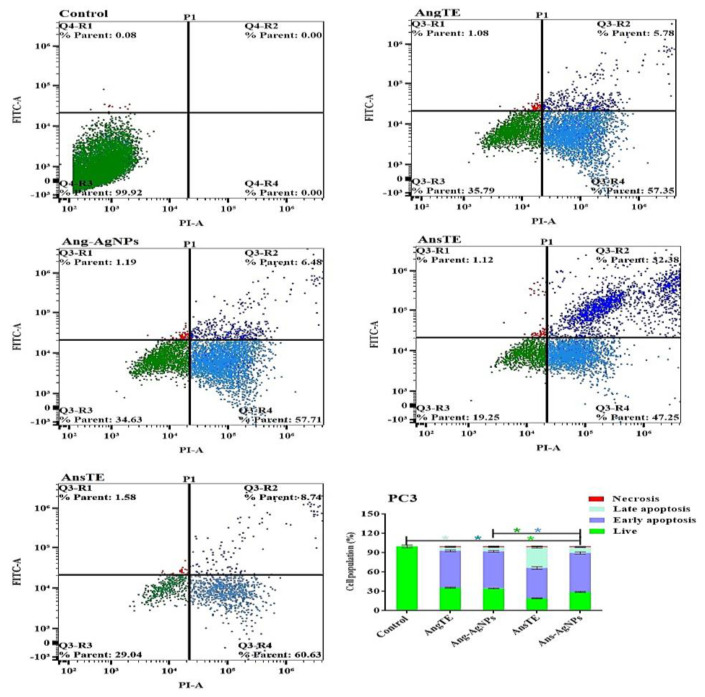
Apoptosis/necrosis assessment for the extracts, AngTE, AnsTE, and their nanoparticles, Ang-AgNPs, and Ans-AgNPs, respectively, against PC3 subjected to previous treatment for 48 h, and apoptosis/necrosis quantified using flow cytometry. Data are presented as the mean ± SD; n = 3 One-way ANOVA was used to test for statistical difference (* *p* < 0.05).

**Figure 13:**
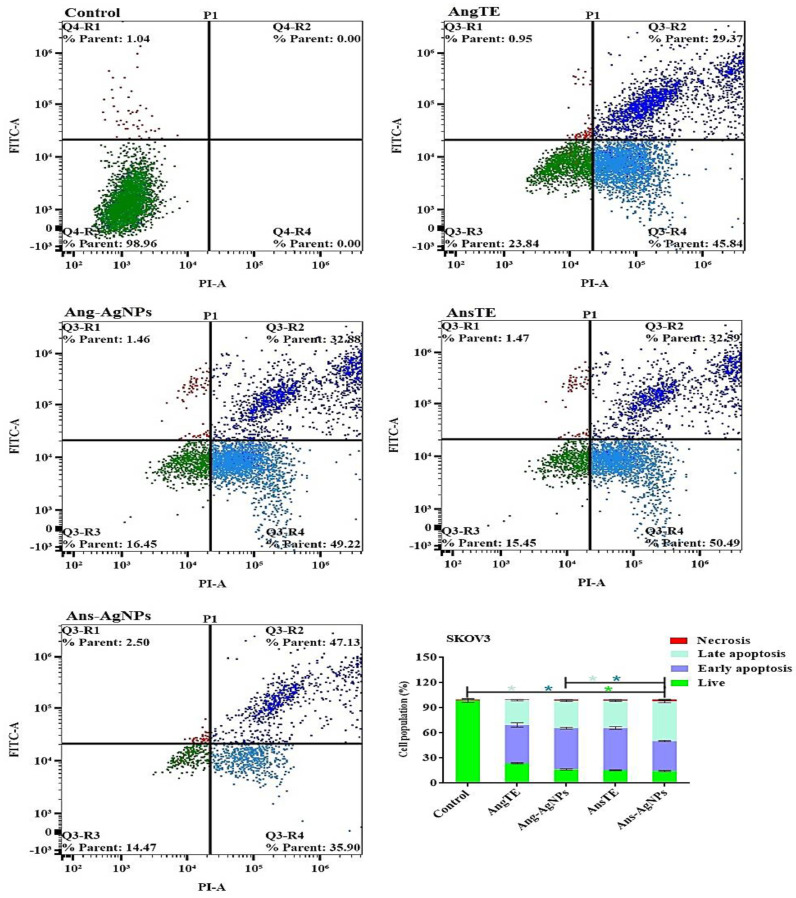
Apoptosis/necrosis assessment of the extracts, AngTE, AnsTE, and their nanoparticles, Ang-AgNPs, and Ans-AgNPs, respectively, against SKOV3 subjected to previous treatment for 48 h, and apoptosis/necrosis quantified using flow cytometry. Data are presented as the mean ± SD; n = 3; One-way ANOVA was used to test for statistical difference (* *p* < 0.05).

**Figure 14:**
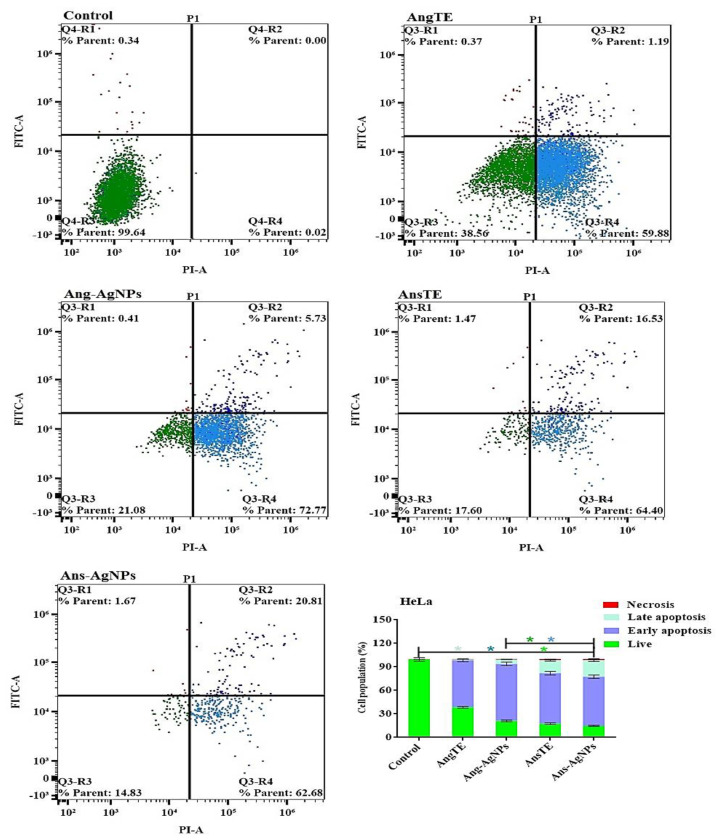
Apoptosis/necrosis assessment of the extracts, AngTE, AnsTE, and their nanoparticles, Ang-AgNPs, and Ans-AgNPs, respectively, against HeLa subjected to previous treatment for 48 h, and apoptosis/necrosis quantified using flow cytometry Data are presented as the mean ± SD; n = 3; One-way ANOVA was used to test for statistical difference (* *p* < 0.05).

In PC3, SKOV3, and HeLa cells, annexin V-FITC/PI staining by a flow cytometer was used to distinguish between cells undergoing apoptosis (programmed cell death) and cells dying via necrosis (non-programmed cell death). There was an increase in the population of apoptotic cells in PC3 cells after treatment with AnsTE of 79.63 ± 3.3%, (Figure 12), as well as in SKOV3 after treatment with AnsTE of 83.1 ± 2.2% and Ans-AgNPs with apoptotic populations of 83.03 ± 2.04, compared to the cell control (Figure 13). Prolonged exposure of HeLa cells to AnsTE and Ans-AgNPs for 48 h significantly induced the number of apoptotic cells by 80.93 ± 3.4% and 83.49 ± 3.7%, respectively (Figure 14), compared to the control cells. In conclusion, AnsTE used at pre-calculated IC_50_ induced a significant apoptotic effect against all tumor cells (PC3, SKOV3, and HeLa).

### 2.4. Docking Study of Bioactive Compounds

At the catalytic site of Bcl-2, the 2D and 3D ligand interaction plots of the identified phytochemical substances are carefully examined. Figure S2 and Table S4 show the Bcl-2 target protein’s binding mechanism for the eight identified substances and navitoclax (ABT-263) in PDB: 4LVT. Each simulation’s gathered trajectory was statistically analyzed for the protein-ligand contacts, thus identifying the crucial amino acids (ARG143, ASN140, and ASP108) needed for efficient Bcl-2 inhibitors. According to the PDB:4LVT, the identified hit compounds cis-annonacin, motrilin, mosinone A, squamocin L, and squamone primarily form hydrogen-bonding interactions with the binding pocket residues ARG143 and ASN140 through water-bridge interactions. Squamocin G and squamocin, which were shown to be hit compounds, also form a second hydrogen bond with ASP137 and ARG104 in PDB:4LVT, in addition to the first hydrogen bonding contact. 

On the other hand, Figure S3 shows the binding mechanism of the Bcl-2 target protein for the eight identified compounds and the navitoclax analogue in PDB: 4MAN. Two interfacing residues that connect hydrophobically are PHE101 and TYR199. The hydrogen bond produced with GLY142, which was also noted in the counterpart of navitoclax, was another significant interaction. In order for cis-Annonacin and squamocin to function as Bcl-2 inhibitors, they had to make essential hydrogen bonds with the GLY142 amino acid residue. The hydrogen bond formed with ARG104, which was seen in cis-annonacin, squamocin, and motrilin, was another important interaction. Both motrilin and squamocin formed a hydrogen connection with the ASP100 amino acid. When compared to other discovered compounds. 

## 3. Discussion

The chemical profiles of ethanolic extracts of *A. squamosa* and *A. glabra* have been analyzed using UPLC-HR-QTOF/MS/MS to highlight the potential active constituents in the extracts. In total, 114 compounds have been identified in the positive ionization mode, including 77 acetogenins, 20 diterpenes, and 17 miscellaneous compounds. 

The biosynthesis of silver nanoparticles using *A. squamosa* and *A. glabra* fruits extracts displayed high quality, biologically active nano silver particles with substantial cytotoxicities towards PC3 and SKOV3 cell lines. The cytotoxicity of AnsTE and Ans-AgNPs on HeLa and SKOV 3 cells was the most potent, with IC_50_s ranging from 0.001 ± 0.0001 to 1.6 ± 0.1 g/mL, and the toxicity of Ans-AgNPs on PC3 cells, with IC_50_ of 1.7 ± 0.4. However, Ang-AgNPs demonstrated the strongest cytotoxic effect on PC3 and SKOV3 with IC_50_ 2.4 ± 0.3 and 2.8 ± 0.23 µg/mL. In the apoptotic study of the two Annona species and their biogenic nanoparticles. The results obtained using the flow cytometry suggest that there was an increase in the population of apoptotic cells in PC3 cells after treatment with AnsTE of 79.63 ± 3.3% as well as in SKOV3 after treatment with AnsTE of 83.1 ± 2.2% and Ans-AgNPs with apoptotic populations of 83.03 ± 2.04, compared to the cell control.

The microbiological evaluation of the formed nanoparticles indicates that Ans-AgNPs has the highest activity with a lower MIC value (0.093 mg/mL) against *C. albicans* while Ang-AgNPs has recorded a higher activity against *P. aeuroginosa*.

These processes were then followed by a molecular docking study to evaluate the role of the identified major active metabolites towards cancer cell lines, where Bcl-2 target protein’s binding mechanism for the eight major identified compounds was studied. It was revealed that the mosinone a, cis-annonacin, squamocin g, motrilin, squamocin have higher binding capacity than other compounds with a binding score of (−10.87–10.56.)

## 4. Materials and Methods

### 4.1. Plant Materials and Extract Preparation

At the Mazhar Botanic Garden, fresh fruits of *Annona glabra* L. and *Annona squamosa* L. were harvested from trees (Giza, Egypt). The botanical specialist Mrs. Therese Labib of Orman and Qubba Gardens verified the authenticity of the voucher specimens. In a blender, fresh samples were homogenized with 70% ethanol. The extracts were dried in a rotary evaporator (Büchi, Essen, Germany under reduced pressure. For further analysis, the dried ethanolic extracts of *A. glabra* (AngTE) and *A. squamosa* (AnsTE) were kept in airtight, dark containers.

### 4.2. Drugs and Chemicals

The highest analytical grades of chemicals and solvents were used, and they were all purchased from Sigma-Aldrich (St. Louis, MO, USA).

### 4.3. UPLC-QTOF-MS/MS Analysis

The LC-MS/MS experiments were carried out at the Faculty of Pharmacy, Fayoum University, using a 6530 Q-TOF LC/MS (Agilent Technologies, Santa Clara, CA, USA) outfitted with an autosampler (G7129A), a quat. pump (G7104C), and a column comp (G7116A). The extracts were separated on an Agilent Technologies Zorbax RP-18 column (150 mm 3 mm, dp = 2.7 m). The flow rate was 0.23 mL/min, and the injection volume was 2 L. The parameters were adjusted as previously described, and mass spectra were acquired using ESI in both ionization modes. A (water with 0.1% formic acid) and B (acetonitrile with 0.1% formic acid) served as the solvents. The following gradient elution times: 0–6 min; 5–50 min; 55–75 min; isocratic elution of 50% A: 50% B; and 75–140 min, were used for the linear gradient from 50% A: 50% B to 100% B.

### 4.4. Green Synthesis of Ang-AgNPs and Ans-AgNPs

To reduce Ag^+^ ions, in a cool, dark setting, 90 mL of a 1 mM AgNO_3_ aqueous solution was mixed with 10 mL of each of AngTE and AnsTE in separate flasks. This mixture was then left at room temperature in complete darkness overnight. After 12 h for Ans-AgNPs and 17 h for Ang-AgNPs, an opaque-brown solution developed, indicating AgNP growth. The prepared solutions were immediately subjected to UV testing. Centrifugation at 4000 rpm for 30 min, three washings in double-distilled water, and filtering were all performed to obtain pure AgNPs. FTIR, Zeta potential, XRD, HR-TEM, and SEM were used to further analyze the pure AgNPs [25,26,27].

### 4.5. Characterization of AgNPs

#### 4.5.1. UV-Vis Spectroscopy 

After dilution with distilled water, the UV absorbance of the bio-synthesized AgNPs from AngTE and AnsTE were examined separately using a UV-Vis spectrophotometer (Shimadzu, Kyoto, Japan).

#### 4.5.2. FTIR

The various functional groups of the formed AngTE and AnsTE were measured using an FTIR spectrometer (Jasco, Tokyo, Japan) in the 4000–400 cm^−1^ range.

#### 4.5.3. HR-TEM

HR-TEM has been used to investigate the particle structure (shape and size) along with SAED. (JEOL-JEM-1011, Kyoto, Japan) and HR-TEM at 200 kV (JEOL-JEM2100, Kyoto, Japan). Three milliliters of the suspended nanoparticles from each sample of Ans-AgNPs and Ang-AgNPs were separately deposited on the copper grid for HR-TEM analysis and were then left to dry at room temperature for 15 min.

#### 4.5.4. Zeta Potential Analysis

A zeta sizer nano ZN was used to investigate zeta potentials and the homogeneity of AngTE and AnsTE (Malvern Panalytical Ltd., Malvern, UK). Prior to the measurements, an aliquot of each Ans-AgNP and Ang-AgNP was combined with ultra-pure water and subjected to sonication for 15 min.

#### 4.5.5. XRD

An XPERT-PRO-PAN-alytical Powder Diffractometer (PAN-alytical B.V., Almelo, The Netherlands) and a monochromatic radiation source Cu-K radiation (θ = 1.5406) at 45 kV and 30 mA at room temperature were used to evaluate metal nanoparticles using XRD analysis as a surface chemical analysis technique. The calculated silver nano-powder intensity data covered a 2 θ range of 4.01°–79.99°.

#### 4.5.6. SEM 

The morphology of the biosynthesized AgNPs was investigated using SEM (TM 1000, Hitachi, Chiyoda, Japan).

### 4.6. Biological Study

#### 4.6.1. Antibacterial Activity 

##### Determination of Antibacterial Activity of the Extracts

The antimicrobial effects were tested against Gram-positive bacteria (*Staphylococcus aureus*), Gram-negative bacteria (*Escherichia coli* and *Pseudomonas aeruginosa*), and the pathogenic fungus (*Candida albicans*).

##### Minimal Inhibitory Concentration (MIC) Determination

Using sterile 2 mL 96-well plates, the antimicrobial activities were assessed [28]. Then, 100 µL of double-strength Mueller Hinton agar that had been sterilized was placed in each well. To create a concentration sequence from 33 mg/mL to 0.128 mg/mL in (AngTE and AnsTE) and from 12 till 0.02 mg/mL in (Ang-AgNPs and Ans-AgNPs), an additional 50 µL of a mixture of culture medium and plant extract (dissolved in 10% DMSO) was serially diluted into the wells 1–9. Well 10 served as growth control, while well 11 was the DMSO negative control. The MIC value (the lowest concentration that completely inhibited the bacterial growth) was determined for each sample after 24 h of incubation at 37 °C. 

##### Disc Diffusion Assay

A modified Kirby-Bauer disc diffusion method was used to measure antimicrobial activity. Then, 100 μL of bacteria were removed from 10 mL of fresh culture media and the bacterial inoculums were standardized with respect to McFarland standard no. 0.5 with 1.5 × 108 CFU/ml bacterial density counted until they reached a density of about 108 cells/mL [29]. Onto Mueller Hinton (MH) agar, 100 μL of the microbial suspension was applied. An aliquot (10 μL) of each of the Ans-AgNPs and Ang-AgNPs, which were dissolved in 10% DMSO, was pipetted onto an agar surface using a sterile paper disc (Whatman No. 1, 5.5 mm paper disc). A filter disc coated with 10 μL of DMSO (Sigma, St. Louis, MO, USA) served as a negative control, and an antibiotic disc (gentamicin for Gram negative bacteria and vancomycin for Gram positive bacteria) was coated with Ans-AgNPs and Ang-AgNPs. The plates were inverted and incubated at 37 ºC for 18 h. Inhibition zone was determined by measuring the diameter of the clear zone (inhibition of growth) around each disc and recorded in millimeter according to CLSI [30].

#### 4.6.2. In Vitro Cytotoxicity

The American type culture collection (ATCC) provided human cell lines for ovary adenocarcinoma (SKOV3), prostate adenocarcinoma (PC3) and human cervical cancer (HeLa). A humidified, 5% (*v*/*v*) CO_2_ atmosphere was used to culture the cells at 37 °C in RPMI-1640 supplemented with (100 g/mL); penicillin (100 units/mL); and heat-inactivated fetal bovine serum (10% *v*/*v*) [31].

##### Cytotoxicity Assay

Using the sulphorhodamine B (SRB) assay, the cytotoxicity of the AngTE, AnsTE, Ang-AgNPs, and Ans-AgNPs against (HeLa, PC-3, and SKOV3) human tumor cells was assessed. Before being treated with the AngTE, AnsTE, Ang-AgNPs, and Ans-AgNPs, cells that were growing at 80% confluency, trypsinized and cultured in a 96-well tissue culture plate for 24 h. Cells were subjected to six different doses of each chemical (0.01, 0.1, 1, 10, and 1000 g/mL) with untreated cells added as a control. Before the cells were fixed with TCA (10% *w*/*v*) for an hour at 4 °C, they were exposed to the concentrations for 72 h. After multiple washings, cells were stained with a 0.4% (*w*/*v*) SRB solution for 10 min in the dark. The surplus stain was eliminated using 1% (*v*/*v*) glacial acetic acid. The SRB-stained cells were dissolved in Tris-HCl buffer after drying overnight. A microplate reader was used to gauge the color intensity at 540 nm. Sigma Plot 12.0 software was used to examine the association between each tumor cell line’s viability percentage and compound concentrations in order to determine the IC_50_ (drug dose that reduces survival to 50%) [31].

##### Cell Cycle Analysis

The IC_50_ values for AngTE, AnsTE, Ang-AgNPs, and Ans-AgNPs were pre-calculated and administered to (HeLa, PC-3, and SKOV3) cells for 48 h. The cells were then fixed in ice-cold 60% ethanol at 40 C and trypsinized before being washed twice in phosphate-buffered saline. After being resuspended, the cells were incubated for 15 min in 500 L of Cell Signaling Technology’s (CST) propidium iodide with RNase staining buffer. In order to evaluate the data from 10,000 cells and the distribution of cell cycle phases for each sample, FACS analysis was completed using a Cytek® Northern Lights 2000 spectral flow cytometer (Cytek Biosciences, Fremont, CA, USA) and SpectroFloTM Software version 2.2.0.3 (Cytek Biosciences, Fremont, CA, USA), both of which are available from the United States [32].

##### Apoptosis Analysis

(HeLa, PC3, and SKOV3) cells were treated for 48 h with AngTE, AnsTE, Ang-AgNPs, and Ans-AgNPs before being trypsinized and subjected to two PBS washes. According to the manufacturer, apoptosis was evaluated using the Annexin V-FITC/PI Apoptosis Detection Kit, Cell Signaling Technology (CST). Briefly, cells were gently mixed with 0.5 mL of binding buffer for 15 min at room temperature in a dark area after being resuspended in 5 μL of Annexin V-FITC, 5 μL of PI (staining solution), and 5 μL of binding buffer. The cells were then subjected to a FACS analysis using a Cytek® Northern Lights 2000 spectral flow cytometer and SpectroFloTM Software version 2.2.0.3 (Cytek Biosciences, Fremont, CA, USA).

### 4.7. Molecular Docking Study

#### 4.7.1. Protein Preparation

The RCSB Protein Data Bank was used to retrieve the Bcl-2 proteins’ structures, which have resolutions of 2.04 and 2.07 (PDB IDs: 4LVT and 4MAN). The system was optimized, the partial charges were computed, and the enzymes were 3D protonated (Figure S2) [33].

#### 4.7.2. Validation of the Molecular Docking Method

The docking methods and parameters were validated to make sure that the ligand orientations derived from the molecular docking simulations are accurate and that the potential binding modes of ligands are reasonable. Using the (MOE, 2019, Montreal, QC, Canada) software (https://www.chemcomp.com/Products.htm), re-docking for co-crystallized ligands in a target protein’s active site after they have been eliminated from Bcl-2 receptor proteins was observed, and a comparison of the results of the final docking (binding mode, hydrogen bond interactions and docking score) was made. The X-ray bioactive conformer of navitoclax and navitoclax analogue was then aligned with the best fitted pose discovered during the docking run [34]. With RMSD = 1.35 and 1.70 for PDB:4LVT and PDB:4MAN (https://www.rcsb.org/structure/4MAN*)*, respectively, the alignment demonstrated good agreement between them, demonstrating the capability of the used docking protocol to retrieve legitimate docking poses. If the returned RMSD value is 3, the method is deemed successful. (Figure S3).

#### 4.7.3. Ligand Preparation

Test ligand structures of eight phytochemical compounds derived from *A. squamosa* and *A. glabra* extracts, navitoclax (ABT-263) and navitoclax analog (with Indole) are prepared for docking by applying the following steps:

(1) ChemBioDraw Ultra 12.0 (CambridgeSoft Corporation, Cambridge, UK) as used to create the target molecules, which were then copied to MOE. (2) The compounds were protonated in three dimensions. (3) The structures were energy minimized to a gradient of 0.5 by the (MMff94x) Merk Molecular Force Field. (4) The force field partial charges for each molecule were determined. (5) Each molecule underwent a stochastic conformational analysis using default parameters, with the results being saved into a different conformational database. (6) To dock into the active site of the androgen receptor, the most stable conformers of each molecule were recorded in a different database.

#### 4.7.4. Molecular Docking Calculations

The most stable conformers were docked using MOE-dock using flexible ligand-rigid receptors as follows: (1) The receptor atoms are defined as (receptor + solvent). (2) The placement site was defined as (ligand atoms). (3) Ligand atoms were retrieved from the conformational database of the target compounds’ least energetic conformers. (4) The placement method was modified to (Alpha triangle). (5) The London dG scoring function was used to estimate the ligand’s binding free energy from a given pose. Finally, the best scoring poses of the docked compounds were identified, and the complexes’ protein-ligand interactions were investigated. 

## 5. Conclusions

To the best of our knowledge, this is the first comprehensive study to compare the metabolic profiles of *A. squamosa* and *A. glabra* fruits using UPLC-HR-QTOF/MS/MS and correlate them with their cytotoxic and antimicrobial activities using molecular docking simulations. Our results revealed that the silver nanoparticles of both species formed a good synergistic effect in combination with gentamicin against *P. aeruginosa* and *E. coli*, respectively. Moreover, the formed nanoparticles potentiated the cytotoxicity of the total extracts against PC3 and SKOV3 cell lines, so the formed nanoparticles are excellent candidates for the treatment of skin infections in patients with ovarian or prostatic cancers.

## Figures and Tables

**Table 1:** Synergistic activity of Ans-AgNPs (4B/FM) and gentamicin against the bacterial species.

	Zone of Inhibition			
	Ang-AgNPs (mm)	Ang-AgNPs *+* Gentamicin (mm)	Gentamicin (mm)	Reference Zone of Inhibition
*Pseudomonas* *aeruginosa*	10	20	14	15
*E. coli*	10	19	14	15

**Table 2:** Synergistic activity of Ans-AgNPs (4D/FM) and Gentamicin against the bacterial species.

	Zone of Inhibition			
	Ans-AgNPs (mm)	Ans-AgNPs + Gentamicin (mm)	Gentamicin	Reference Zone of Inhibition in CLSI
*Pseudomonas aeruginosa*	13	20	12	15
*E. coli*	12	15	15	15

**Table 3:** Synergistic activity of Ans-AgNPs (4D/FM) and vancomycin against *S. aureus*.

	Zone of Inhibition		
	Ans-AgNPs	Ans-AgNPs + Vancomycin	Vancomycin
*Staphylococcus aureus*	15	12	17

## Data Availability

Data are contained within the article and Supplementary Material.

## Supplementary Materials

The following are available online at https://www.mdpi.com/article/10.3390/ph15111354/s1, Figure S1. UPLC/MS chromatograms of identified constituents in the ethanolic extracts of *A. squamosa* (A) and *A. glabra* (B) fruits detected in positive ionization mode. Figure S2. (A) Bcl-2 receptor (PDB: 4LVT) (Left) and PDB ID:4MAN) (Right), (B) Bcl-2 Navitoclax (ABT-263) Complex (Left) and Bcl-2-Navitoclax Analog (with Indole) Complex (Right). Figure S3. The alignment between the X-ray bioactive conformer of the navitoclax and its analog (colored in yellow) and the redocked pose of the same compound (colored in cyan) at Bcl-2 receptor binding site proteins. Table S1: Compounds identified in the extracts of *A. glabra* (AngTE) and *A. squamosa* (AnsTE) fruits, by UPLC-QTOF-MS/MS (positive mode). Table S2. Results of the minimum inhibitory concentration (MIC) of the extracts. Table S3. The IC_50_ (µg) of the ethanolic extracts of *A. squamosal* and *A. glabra*, and their nanoparticles against different human solid tumor cells. Table S4. Molecular docking results of major identified compounds from the extracts of *A. squamosal* and *A. glabra*.

## References

[1] Biba V., Amily A., Sangeetha S., Remani P. (2014). Anticancer, antioxidant and antimicrobial activity of annonaceae family. World J. Pharm. Pharm. Sci..

[2] Nugraha A.S., Damayanti Y.D., Wangchuk P., Keller P.A.J.M. (2019). Anti-infective and anti-cancer properties of the *Annona* species: Their ethnomedicinal uses, alkaloid diversity, and pharmacological activities. Molecules.

[3] Chen Y.-Y., Ma C.-Y., Wang M.-L., Lu J.-H., Hu P., Chen J.-W., Li X., Chen Y.J.N.P.R. (2020). Five new ent-kaurane diterpenes from *Annona squamosa* L. Pericarps. Nat. Prod. Res..

[4] Dev A.A., Joseph S.M. (2021). Anticancer potential of *Annona genus*: A detailed review. J. Indian Chem. Soc..

[5] Feriani A., Tir M., Gómez-Caravaca A.M., del Mar Contreras M., Talhaoui N., Taamalli A., Se-gura-Carretero A., Ghazouani L., Mufti A., Tlili N. (2020). Hplc-dad-esi-qtof-ms/ms profiling of zy-gophyllum album roots extract and assessment of its cardioprotective effect against deltame-thrin-induced myocardial injuries in rat, by suppression of oxidative stress-related inflammation and apoptosis via nf-κb signaling pathway. J. Ethnopharmacol..

[6] Hamed A.R., El-Hawary S.S., Ibrahim R.M., Abdelmohsen U.R., El-Halawany A.M. (2021). Identification of chemoprevetive components from halophytes belonging to aizoaceae and cactaceae through lc/ms—Bioassay guided approach. J. Chromatogr. Sci..

[7] Macintyre L., Zhang T., Viegelmann C., Martinez I.J., Cheng C., Dowdells C., Abdelmohsen U.R., Gernert C., Hentschel U., Edrada-Ebel R. (2014). Metabolomic tools for secondary metabolite discovery from marine microbial symbionts. Mar. Drugs.

[8] Attallah N.G., Elekhnawy E., Negm W.A., Hussein I.A., Mokhtar F.A., Al-Fakhrany O.M. (2022). In vivo and in vitro antimicrobial activity of biogenic silver nanoparticles against staphylococcus aureus clinical isolates. Pharmaceuticals.

[9] Thakkar K.N., Mhatre S.S., Parikh R.Y. (2010). Biological synthesis of metallic nanoparticles. Nanomed. Nanotechnol. Biol. Med..

[10] Pearce A., Haas M., Viney R., Pearson S.-A., Haywood P., Brown C., Ward R. (2017). Incidence and severity of self-reported chemotherapy side effects in routine care: A prospective cohort study. PLoS ONE.

[11] Majeed H., Gupta V. (2021). Adverse Effects of Radiation Therapy.

[12] Issa M.Y., Mohsen E., Younis I.Y., Nofal E.S., Farag M.A. (2020). Volatiles distribution in jasmine flowers taxa grown in egypt and its commercial products as analyzed via solid-phase microextraction (spme) coupled to chemometrics. Ind. Crops Prod..

[13] Jang S.J., Yang I.J., Tettey C.O., Kim K.M., Shin H.M. (2016). In-vitro anticancer activity of green synthesized silver nanoparticles on mcf-7 human breast cancer cells. Mater. Sci. Eng. C.

[14] Langsrud S., Sidhu M.S., Heir E., Holck A.L. (2003). Bacterial disinfectant resistance—A challenge for the food industry. Int. Biodeterior. Biodegrad..

[15] Ahmed S., Ahmad M., Swami B.L., Ikram S. (2016). A review on plants extract mediated synthesis of silver nanoparticles for antimicrobial applications: A green expertise. J. Adv. Res..

[16] Shiekh K.A., Olatunde O.O., Zhang B., Huda N., Benjakul S.J.F.C. (2021). Pulsed electric field assisted process for extraction of bioactive compounds from custard apple (*Annona squamosa*) leaves. Food Chem..

[17] Bhardwaj R., Pareek S., Sagar N., Vyas N. (2020). Bioactive compounds of *Annona*. Bioactive Compounds in Underutilized Fruits and Nuts.

[18] Ma C.-Y., Li J.-H., Li X., Liu X., Chen J.-W. (2019). Eight new cytotoxic annonaceous acetogenins from the seeds of *Annona squamosa*. Chin. J. Nat. Med..

[19] Nhiem N.X., Hien N.T.T., Tai B.H., Anh H.L.T., Hang D.T.T., Quang T.H., Van Kiem P., Van Minh C., Ko W., Lee S.J.B. (2015). New ent-kauranes from the fruits of *Annona glabra* and their inhibitory nitric oxide production in lps-stimulated raw264. 7 macrophages. Bioorganic Med. Chem. Lett..

[20] Chen Y., Chen J.-w., Wang Y., Xu S.-s., Li X. (2012). Six cytotoxic annonaceous acetogenins from *Annona squamosa* seeds. Food Chem..

[21] Chen C.-Y., Chang F.-R., Cho C.-P., Wu Y.-C. (2000). Ent-kaurane diterpenoids from *Annona glabra*. J. Nat. Prod..

[22] Bonneau N., Baloul L., ba Ndob I.B., Senejoux F., Champy P. (2017). The fruit of *Annona squamosa* L. As a source of environmental neurotoxins: From quantification of squamocin to annotation of an-nonaceous acetogenins by lc–ms/ms analysis. Food Chem..

[23] Avula B., Bae J.-Y., Majrashi T., Wu T.-Y., Wang Y.-H., Wang M., Ali Z., Wu Y.-C., Khan I.A. (2018). Targeted and non-targeted analysis of annonaceous alkaloids and acetogenins from *asimi-na* and *Annona* species using uhplc-qtof-ms. J. Pharm. Biomed. Anal..

[24] Bykkam S., Ahmadipour M., Narisngam S., Kalagadda V.R., Chidurala S.C. (2015). Extensive studies on x-ray diffraction of green synthesized silver nanoparticles. Adv. Nanopart..

[25] Tarannum N., Gautam Y.K. (2019). Facile green synthesis and applications of silver nanoparticles: A state-of-the-art review. RSC Adv..

[26] Desai R., Mankad V., Gupta S.K., Jha P.K. (2012). Size distribution of silver nanoparticles: Uv-visible spectroscopic assessment. Nanosci. Nanotechnol. Lett..

[27] Varadavenkatesan T., Vinayagam R., Selvaraj R. (2017). Structural characterization of silver nanoparticles phyto-mediated by a plant waste, seed hull of vigna mungo and their biological applications. J. Mol. Struct..

[28] Wiegand I., Hilpert K., Hancock R.E. (2008). Agar and broth dilution methods to determine the minimal inhibitory concentration (mic) of antimicrobial substances. Nat. Protoc..

[29] Pfaller M.A., Burmeister L., Bartlett M., Rinaldi M. (1988). Multicenter evaluation of four methods of yeast inoculum preparation. J. Clin. Microbiol..

[30] Humphries R.M., Ambler J., Mitchell S.L., Castanheira M., Dingle T., Hindler J.A., Koeth L., Sei K. (2018). Clsi methods development and standardization working group best practices for evaluation of antimicrobial susceptibility tests. J. Clin. Microbiol..

[31] Mahmoud A.M., Al-Abd A.M., Lightfoot D.A., El-Shemy H.A. (2012). Anti-cancer characteristics of mevinolin against three different solid tumor cell lines was not solely p53-dependent. J. Enzyme Inhib. Med. Chem..

[32] Nunez R. (2001). DNA measurement and cell cycle analysis by flow cytometry. Curr. Issues Mol. Biol..

[33] Vafaei S., Sadat Shandiz S.A., Piravar Z. (2020). Zinc-phosphate nanoparticles as a novel anticancer agent: An in vitro evaluation of their ability to induce apoptosis. Biol. Trace Elem. Res..

[34] Wakui N., Yoshino R., Yasuo N., Ohue M., Sekijima M. (2018). Exploring the selectivity of inhibitor complexes with bcl-2 and bcl-xl: A molecular dynamics simulation approach. J. Mol. Graph. Model..

