# Supplementary material for: Green Biosynthesis of Silver Nanoparticles Using Annona glabra and Annona squamosa Extracts with Antimicrobial, Anticancer, Apoptosis Potentials, Assisted by In Silico Modeling, and Metabolic Profiling
Source: Pharmaceuticals (Basel). 2022 Nov 2;15(11):1354. doi: 10.3390/ph15111354 (PMC9692630; doi:10.3390/ph15111354)
Supplement: Supplementary file 1 [file pharmaceuticals-15-01354-s001.zip › pharmaceuticals-1975483-supplementary.pdf]

Supplementary materials

Green biosynthesis of silver nanoparticles using *Annona glabra* and *Annona squamosa* extracts with antimicrobial, anticancer, apoptosis potentials, Assisted by *in Silico* Modeling, and Metabolic Profiling

Fatma A. Mokhtar[1*⫛], Nabil M. Selim[2*⫛], Seham S. Elhawary [2], Soha R. Abd El Hadi[3], Mona H. Hetta[4], Marzough A albalawi[5], Ali A. Shati[6], Mohammad Y. Alfaifi[6], Serag Eldin I. Elbehairi[6,7], Lamia I. Fahmy[8], Rana M. Ibrahim[2]

[1] Department of Pharmacognosy, Faculty of Pharmacy, Al Salam University, kafr alzayat, Al Gharbia, Egypt

[2] Pharmacognosy Department, Faculty of Pharmacy, Cairo University, Kasr-El-Ainy Street, 11562, Cairo, Egypt

[3] Department of Pharmaceutical Chemistry, Faculty of Pharmacy, Egyptian Russian University, Badr City, Cairo, Egypt

[4] Department of Pharmacognosy, Faculty of Pharmacy, Fayoum University, Fayoum, 63514, Egypt

[5] Department of Chemistry, Alwajh college, University of Tabuk, Saudi Arabia

[6] King Khalid University, Faculty of Science, Biology Department, Abha 9004, Saudi Arabia

[7] Cell Culture Lab, Egyptian Organization for Biological Products and Vaccines (VACSERA Holding Company), 51 Wezaret El-Zeraa St., Agouza, Giza, Egypt

[8] Department of microbiology and immunology, Faculty of Pharmacy, October University for Modern Sciences and Arts (MSA), Giza, Egypt

*Corresponding authors;

Fatma A. Mokhtar: Fatma.Mokhtar@Sue.edu.eg

Nabil M. Selim; nabil.selim@pharma.cu.edu.eg, orchid ID, 0000-0001-6252-000X

⫛ These authors contributed equally to this work.

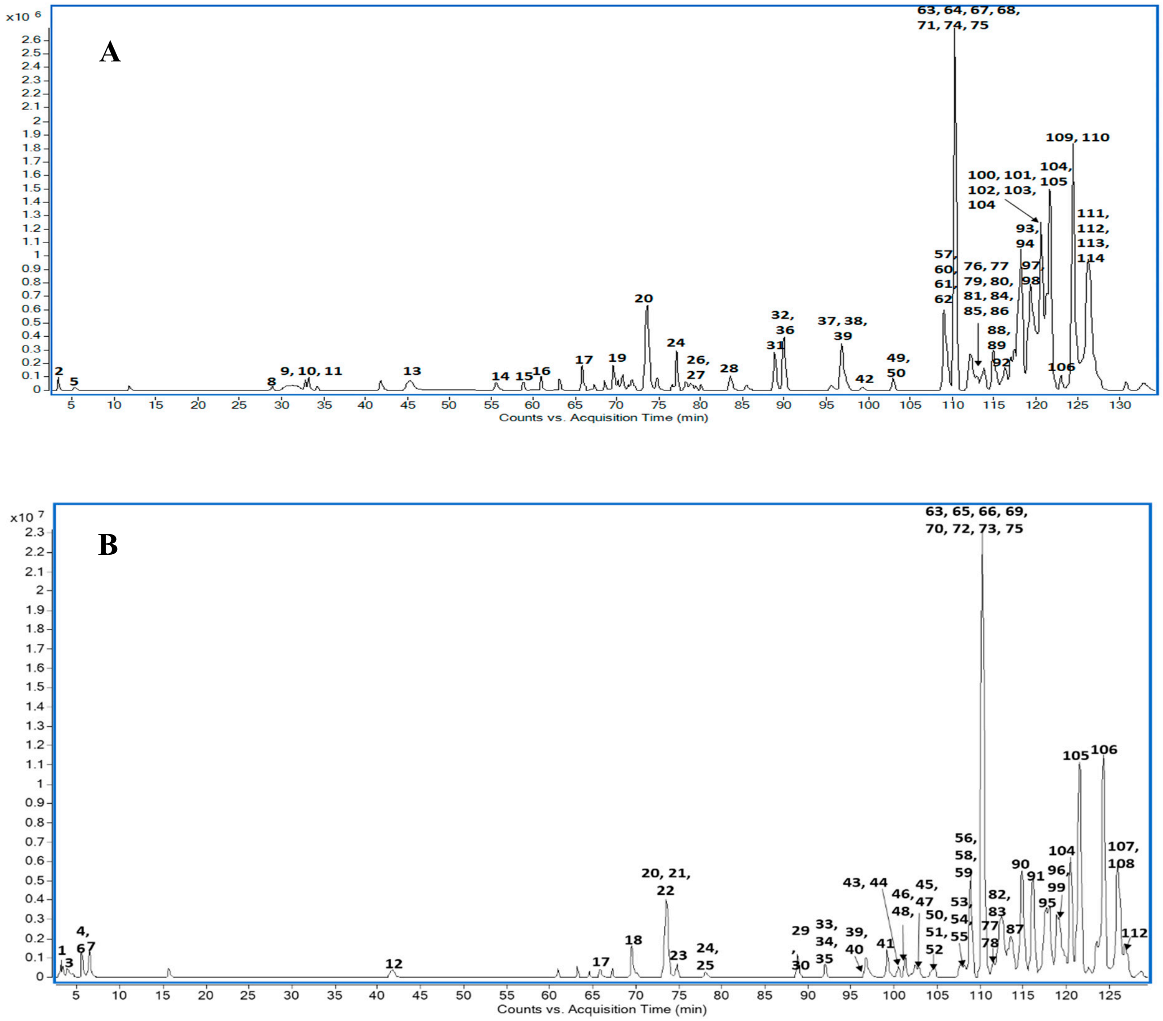

Figure S1. UPLC/MS chromatograms of identified constituents in the ethanolic extracts of *A. squamosa* (A) and *A. glabra* (B) fruits detected in positive ionization mode.

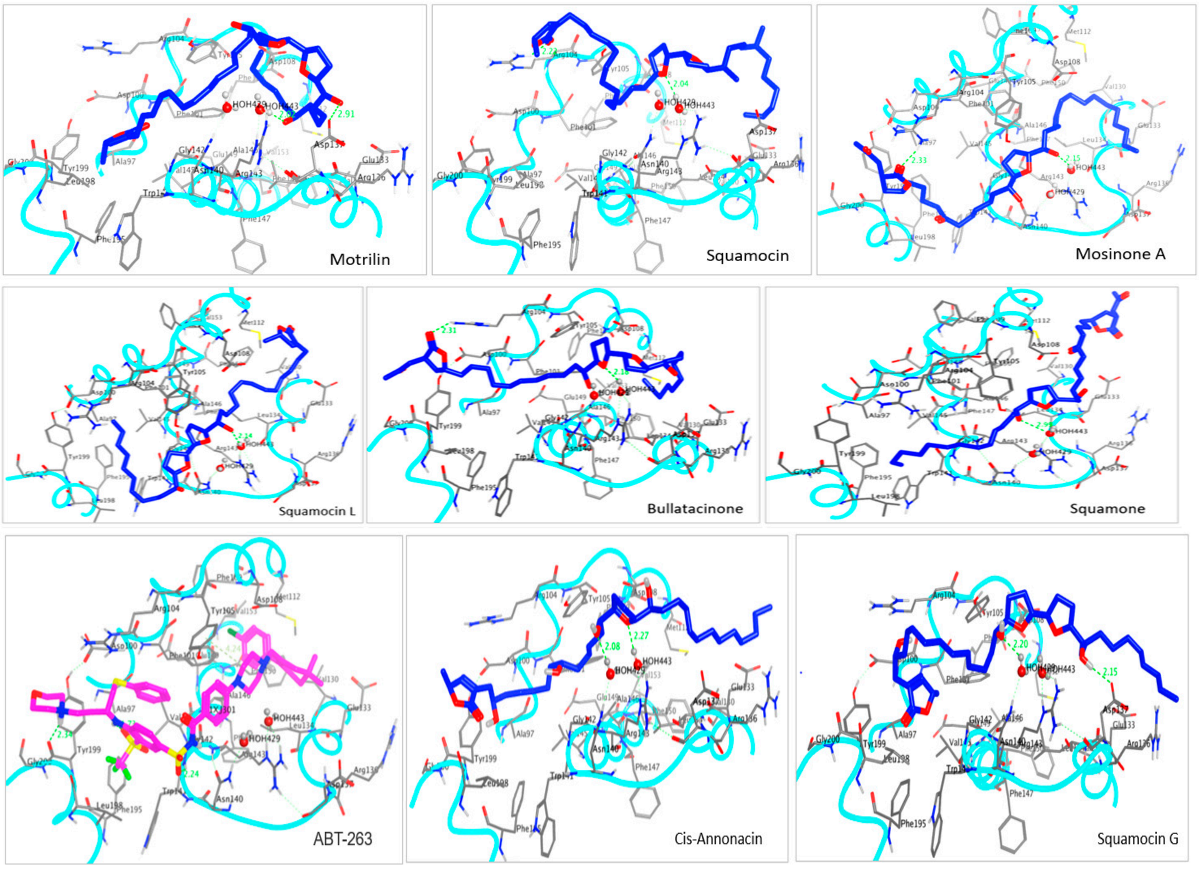

Figure S2. Visualization of interactions between the Bcl-2 receptor (4-LVT) and ABT-263, cis-annonacin, squamocin G, motrilin, squamocin, mosinone A, Squamocin L, bullatacinone, squamone, showed comparable or better binding energy and binding modes.

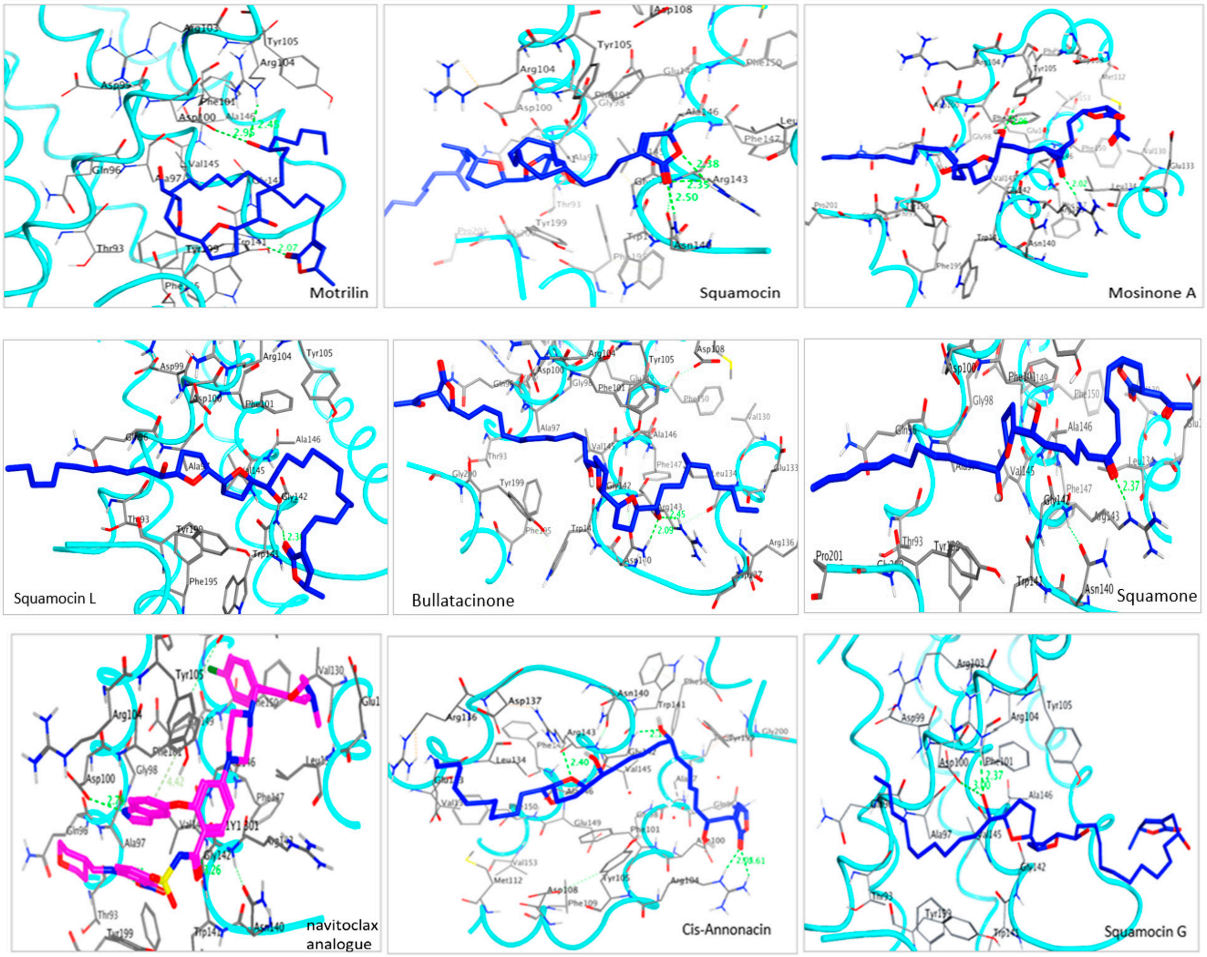

Figure S3. Visualization of interactions between the Bcl-2 receptor (4-MAN) and motrilin, squamocin, mosinone A, navitoclax analogue, cis-annonacin, squamocin G, squamocin L, bullatacinone, squamone, showed comparable or better binding energy and binding modes.

Table S1. Compounds identified in the extracts of *A. glabra* (AngTE) and *A. squamosa* (AnsTE) fruits, by UPLC-QTOF-MS/MS (positive mode).

Peak no.	R_T (min)	Indicated Molecular ion (m/z)		Error ppm	Elemental composition	Identification	MS/MS (*m/z*)	Source
		$(M+H)^+$	$(M+Na)^+$					
1	3.08		219.0266	1.41	$C_9H_9O_5^+$	Trihydroxycinnamic acid	203, 181, 147, 134, 112, 104	AngTE
2	3.19	183.086	205.0677	0.56	$C_6H_{15}O_6^+$	Sorbitol	148, 118, 96, 74	AnsTE
3	3.23		235.0206	4.62	$C_9H_9O_6^+$	Pentahydroxy cinnamaldehyde	222, 219, 203, 189, 184	AngTE
4	4.09		217.0681	1.23	$C_7H_{15}O_6^+$	Methyl inositol	169, 157, 138, 129, 118	AngTE
5	5.21	88.0758		-0.53	$C_4H_{10}NO^+$	2-Hydroxypyrrolidine	74, 60	AnsTE
6	5.61		215.0516	4.03	$C_6H_9O_7^+$	Citric acid	186, 169, 152, 140, 130, 122, 96, 82	AngTE
7	6.42		231.0839	-0.11	$C_8H_{17}O_6^+$	Dimethyl inositol	226, 209, 186, 169, 163, 145, 129, 112	AngTE
8	32.69	195.101		0.43	$C_{11}H_{15}O_3^+$	Methoxyeugenol	174, 169, 112, 96, 82	AnsTE
9	34.1	328.153		1.75	$C_{19}H_{22}NO_4^+$	Norcorydine	276, 257, 234, 221, 197, 185, 174, 169, 157, 147	AnsTE
10	34.1	139.038		2.41	$C_7H_7O_3^+$	Salicylic acid	112, 84	AnsTE
11	34.1		141.05	6.89	$C_4H_7O_4^+$	Succinic acid	112, 96	AnsTE
12	41.57	323.169	345.1511	2.47	$C_{14}H_{27}O_8^+$	Pachybiose	276, 231, 197, 147, 120, 96	AngTE
13	45.31	107.070	129.0519	-0.22	$C_4H_{11}O_3^+$	Butanetriol	96, 84	AnsTE
14	55.72		217.1045	1.47	$C_8H_{19}O_5^+$	Dimethyl rhamnitol	174, 169, 112, 96, 82	AnsTE
15	58.85	255.267		3.12	$C_{17}H_{35}O^+$	Dimethyl pentadecanone	234, 169, 151, 129, 96, 74, 64	AnsTE
16	60.87	235.116	257.099	1.78	$C_{10}H_{19}O_6^+$	Dihydroxy dimethyloctanedioic acid	197, 120, 96	AnsTE

17	65.92	391.248		2.60	$C_{23}H_{35}O_5^+$	**16-acetoxy-*ent*-kauran-19-al-17-methyl ester (Annoglabasin D)**	331, 300, 272, 233, 197, 169, 120	AnsTE, AngTE
18	70.26	353.228		-5.02	$C_{20}H_{33}O_5^+$	7,16,17-trihydroxy-ent-kauran-19-oic acid	309, 295, 265, 236, 213, 195, 185, 176, 156, 144, 129	AngTE
19	70.68	319.226		1.66	$C_{20}H_{31}O_3^+$	19-Oxo-17-kauranoic acid	285, 253, 197, 167, 120	AnsTE
20	73.57	303.230		1.75	$C_{20}H_{31}O_2^+$	Ent-kaur-16-en-19-oic acid	242, 167, 151, 129	AnsTE, AngTE
21	73.71	379.242		-14.9	$C_{22}H_{35}O_5^+$	16-acetoxy-19-nor-ent-kauran-4-ol-17-methyl ester (Annoglabasin F)	361, 302, 271, 243, 181, 149	AngTE
22	74.05	225.151		11.4	$C_{13}H_{21}O_3^+$	Blumenol A	159, 143	AngTE
23	74.91	337.233		-10.6	$C_{20}H_{33}O_4^+$	16,17-Dihydroxy-ent-kauran-19-oic acid	321, 296, 263, 205, 181, 158, 134	AngTE
24	77.24	303.231		0.7	$C_{20}H_{31}O_2^+$	17-Hydroxy-15-kauren-19-al	289, 248, 221, 193, 167, 144, 129	AnsTE, AngTE
25	78.04	335.219		5.26	$C_{20}H_{31}O_4^+$	16 Hydro-ent-kauran-17,19-dioic acid	307, 279, 242, 213, 181, 149	AngTE
26	79.85	289.251		3.51	$C_{20}H_{33}O^+$	Ent-kaur-16-en-19-ol	271, 249, 208, 177, 161	AnsTE
27	81.48		373.2349	-8.72	$C_{21}H_{35}O_4^+$	Annosquamosin D	352, 333, 301, 278, 256, 239, 17, 197, 185, 157, 144	AnsTE
28	85.83		317.2103	-3.91	$C_{18}H_{31}O_3^+$	Squamostanal A	305, 285, 255, 217, 197, 185, 167, 120	AnsTE
29	88.75	319.223		9.63	$C_{20}H_{31}O_3^+$	16-Hydro-19-al-ent-kauran-17-oic acid	297, 253, 186, 144	AngTE
30	88.95	639.481	661.4632	3.81	$C_{37}H_{67}O_8^+$	Squamostatin B	621, 603, 585, 567, 395, 319, 297, 279, 186, 144	AngTE
31	89.87	287.203		-0.57	$C_{20}H_{31}O^+$	16-Kauren-19-al	269, 243, 233, 213, 197, 186, 167, 144, 125	AnsTE

32	91.28	305.247		-0.13	$C_{20}H_{33}O_2^+$	17-Hydroxy-16-kauran-19-al	287, 235, 185, 157, 120, 105	AnsTE
33	91.86	331.225		-3	$C_{21}H_{31}O_3^+$	Methyl ent-7,15-dihydroxy-kaur-16-en-19-oate	302, 277, 255, 217, 197, 186	AngTE
34	91.88	639.481	661.4633	2.89	$C_{37}H_{67}O_8^+$	27-Hydroxybullatacin	393, 352, 339, 302, 277, 255, 186, 167, 144, 124	AngTE
35	92.05	639.481		1.9	$C_{37}H_{67}O_8^+$	Cherimolin-2	409, 352, 321, 281, 250, 217, 185, 144	AngTE
36	92.6	639.482		-0.84	$C_{37}H_{67}O_8^+$	Squamotin D	401, 352, 321, 281, 203, 167	AnsTE
37	96.53	639.482	661.465	1.3	$C_{37}H_{67}O_8^+$	Squamostanin A	601, 579, 507, 463, 401, 352, 323, 301, 279, 205,167, 137	AnsTE
38	96.84	639.482	661.464	5	$C_{37}H_{67}O_8^+$	Squamostanin B	579, 507, 463, 352, 317, 301, 205, 185, 167, 137	AnsTE
39	96.94	639.482	661.4643	2.95	$C_{37}H_{67}O_8^+$	Squamostanin C	601, 579, 507, 463, 401,352, 323, 301, 205,167, 137	AnsTE, AngTE
40	97.03	611.451		2.59	$C_{35}H_{63}O_8^+$	Annoglaxin	409, 352, 295, 186, 167, 144, 125	AngTE
41	99.16	639.481	661.4635	2.83	$C_{37}H_{67}O_8^+$	Glabracin A	449, 431, 413, 379, 361, 311, 309, 293, 291, 275, 241, 223, 215, 171, 141	AngTE
42	99.27	639.485		-4.64	$C_{37}H_{67}O_8^+$	Annosquatin IV	401, 352, 301, 217, 124	AnsTE
43	100.47	639.480	661.4626	4.12	$C_{37}H_{67}O_8^+$	Glabracin B	449, 431, 413, 379, 361, 311, 309, 293, 291, 275, 241, 223, 215, 171, 141	AngTE
44	101.38	639.480	661.4627	3.88	$C_{37}H_{67}O_8^+$	Squamostatin C	613, 401, 352, 339, 331, 301, 279, 250, 225, 186, 167, 144	AngTE

45	102.6		661.465	-0.56	$C_{37}H_{67}O_8^+$	Annosquatin I	617, 595, 401, 352, 301, 217, 124	AngTE
46	102.89	595.454	617.4368	3.4	$C_{35}H_{63}O_7^+$	Isoannonacinone	401, 352, 317, 217, 167, 144	AngTE
47	103.1	595.457	617.4404	-1.69	$C_{35}H_{63}O_7^+$	Molvizarin	512, 443, 401, 352, 309, 285, 250, 167, 96, 64	AngTE
48	103.67	321.239		-8.3	$C_{20}H_{33}O_3^+$	16-Hydroxy-ent-kauran-19-oic acid (Annoglabasin E)	289, 271, 243, 229, 181, 151, 126	AngTE
49	103.77		331.2249	1.8	$C_{19}H_{33}O_3^+$	Annosquamosin B	285, 258, 232, 182, 137	AnsTE
50	104.61	639.481	661.4627	3.34	$C_{37}H_{67}O_8^+$	Squamostanin D	613, 401, 352, 319, 279, 217, 167, 144, 124	AnsTE, AngTE
51	104.64	597.470		4.35	$C_{35}H_{65}O_7^+$	Annonacin	485, 352, 331, 225, 167, 144	AngTE
52	104.93	591.494	613.4787	2.68	$C_{37}H_{67}O_5^+$	Glabrencin A	597, 526, 482, 438, 401, 352, 326, 307, 285, 217, 197, 167, 144	AngTE
53	107.74	347.254		9.51	$C_{22}H_{35}O_3^+$	16-hydro-19-acetoxy-ent-kauran-17-al (Annoglabasin G)	331, 317, 303, 301, 273, 217, 185	AngTE
54	108.25		617.4382	-1.11	$C_{35}H_{63}O_7^+$	parviflorin	377, 352, 307, 279, 197, 167, 144, 124	AngTE
55	108.85	623.486	645.4687	2.31	$C_{37}H_{67}O_7^+$	Glabranin	597, 427, 401, 379, 352, 331, 307, 285, 257, 232, 167, 144, 124	AngTE
56	109	623.490	645.4709	-1.14	$C_{37}H_{67}O_7^+$	Squamotin A	605, 591, 493, 428, 401, 352, 320, 303, 285, 257, 232, 212, 167, 144, 124	AngTE
57	109.26	597.471		-2.12	$C_{35}H_{65}O_7^+$	*cis*-Annonacin	591, 501, 469, 428, 401, 352, 303, 285	AnsTE
58	109.36		619.452	2.5	$C_{35}H_{65}O_7^+$	Glacin A	352, 331, 313, 281, 263, 225, 167, 144	AngTE
59	109.56	623.486	645.4684	2.63	$C_{37}H_{67}O_7^+$	4-Deoxy-6-hydroxysquamotacin	597, 427, 401, 379, 352, 331, 303, 285, 257, 232, 167, 144, 124	AngTE
60	109.85	597.473		0.55	$C_{35}H_{65}O_7^+$	Squamoxinone C	591, 441, 427, 401, 352, 320, 303, 285, 257, 232, 212	AnsTE

61	109.86	597.473		0.55	$C_{35}H_{65}O_7^+$	Squadiolin C	579, 561, 543, 525 427, 401, 352, 303, 257, 212	AnsTE
62	109.86		645.4711	-1.52	$C_{37}H_{67}O_7^+$	Squamosten A	501, 504, 352, 284, 217, 144, 124	AnsTE
63	110.07		645.4713	-1.9	$C_{37}H_{67}O_7^+$	Squamocin C	613, 591, 501, 406, 384, 352, 338, 301, 124	AnsTE, AngTE
64	110.16	597.472		0.33	$C_{35}H_{65}O_7^+$	Isoannonacin	587, 493, 428, 401, 363, 352, 303, 257, 232	AnsTE
65	110.27	623.486	645.4683	2.74	$C_{37}H_{67}O_7^+$	Squamostatin E	331, 323, 303, 294, 257, 232, 167, 144, 124	AngTE
66	110.48		645.4715	-2.01	$C_{37}H_{67}O_7^+$	Annosquatin V	591, 401, 352, 303, 217, 167, 124, 96	AngTE
67	110.96	597.476		-3.01	$C_{35}H_{65}O_7^+$	Annonacin A	493, 428, 401, 352, 303, 257, 232, 185	AnsTE
68	110.96		645.4709	-1.14	$C_{37}H_{67}O_7^+$	Squamocin H	554, 401, 371, 352, 217, 144, 124	AnsTE
69	111.43	305.246		-4.1	$C_{20}H_{33}O_2^+$	16-Hydro-17-hydroxy-ent-kauran-19-al	270, 204, 173, 136	AngTE
70	111.48	363.249		9.65	$C_{22}H_{35}O_4^+$	16α-hydro-19-acetoxy-ent-kauran-17-oic acid (Annoglabasin B)	345, 323, 311, 305, 289, 271, 234	AngTE
71	111.69	595.458		-7.52	$C_{35}H_{63}O_7^+$	Squamone	512, 468, 401, 352, 325, 285, 250, 217, [illegible]	AnsTE
72	111.78		619.4498	3.04	$C_{35}H_{65}O_7^+$	Glacin B	401, 352, 331, 225, 167, 144	AngTE
73	111.88	595.454	617.437	1.22	$C_{35}H_{63}O_7^+$	6-Hydroxydesacetyluvaricin	401, 352, 317, 217, 167, 144	AngTE
74	111.89		613.4816	-2.73	$C_{37}H_{67}O_5^+$	Squamocenin	605, 587, 493, 428, 401, 363, 352, 325, 303, 285, 257, 232, 212, 176, 144	AnsTE
75	111.89		645.4703	0.16	$C_{37}H_{67}O_7^+$	Bullatacinone	613, 591, 540, 496, 401, 384, 352, 331, 301, 285, 205, 124	AnsTE, AngTE

76	112.1		617.4397	-3.95	$C_{35}H_{63}O_7^+$	Bullacin	591, 540, 496, 452, 401, 352, 331, 285, 205, 185, 144, 124	AnsTE
77	112.19	623.486	645.4682	2.84	$C_{37}H_{67}O_7^+$	Squamocin D	401, 352, 331, 313, 281, 180, 144, 124	AnsTE, AngTE
78	112.79		603.4591	-3.13	$C_{35}H_{65}O_6^+$	Corossolin	427, 352, 331, 309, 271	AngTE
79	112.8		617.4393	-0.82	$C_{35}H_{63}O_7^+$	Annonacinone	557, 559, 541, 496, 457, 401, 352, 331, 285, 205	AnsTE
80	112.8	595.458		-0.03	$C_{35}H_{63}O_7^+$	Squamolinone	556, 443, 401, 352, 317, 217, 124	AnsTE
81	113.1	595.457	617.4393	-1.28	$C_{35}H_{63}O_7^+$	Squamocin E	512, 443, 401, 352, 309, 250, 217, 167, 124	AnsTE
82	113.6	623.486	645.4698	1.23	$C_{37}H_{67}O_7^+$	Annonin I	379, 352, 323, 285, 198, 144, 124	AngTE
83	113.6		613.4841	-3.71	$C_{37}H_{67}O_5^+$	Glabrencin B	379, 352, 323, 285, 217, 198, 144, 124	AngTE
84	113.71	639.482	661.4672	-3.71	$C_{37}H_{67}O_8^+$	9-Hydroxyasimicinone	628, 613, 591, 429, 352, 301, 185, 167, 144, 124	AnsTE
85	113.8	595.458	617.4393	-0.03	$C_{35}H_{63}O_7^+$	Mosin B	556, 443, 401, 352, 317, 285, 250, 217, 124	AnsTE
86	114.71	639.484	661.4632	3.41	$C_{37}H_{67}O_8^+$	Annonin XIV	628, 613, 591, 429, 352, 301, 185, 167, 144, 124	AnsTE
87	114.81	623.486	645.4683	2.57	$C_{37}H_{67}O_7^+$	Bullatacin	587, 569, 401, 371, 352, 347, 325, 307, 144, 124	AG TE
88	115.1		617.4404	-1.69	$C_{35}H_{63}O_7^+$	Squamocin B	540, 401, 352, 285, 205, 124	AnsTE
89	115.13		315.2298	1.3	$C_{19}H_{33}O_2^+$	Annosquamosin C	308, 299, 295, 283, 281, 256, 232, 203, 193, 180, 134	AnsTE
90	115.14	623.486	645.469	1.62	$C_{37}H_{67}O_7^+$	Asimicin	583, 401, 352, 325, 285, 144, 124	AG TE

91	115.15		645.4681	2.27	$C_{37}H_{67}O_7^+$	Motrilin	562, 384, 352, 285, 144, 124	AG TE
92	116.1	595.458	617.4393	-1.28	$C_{35}H_{63}O_7^+$	Mosin C	512, 443, 401, 352, 309, 250, 167, 96	AnsTE
93	117.77		601.4455	0.63	$C_{37}H_{61}O_6^+$	Squamocin K	501, 391, 285, 144	AnsTE
94	118.26		645.47	0.08	$C_{37}H_{67}O_7^+$	Squamocin	591, 352, 331, 303, 217, 124	AnsTE
95	118.45		615.4934	5.94	$C_{37}H_{69}O_5$	Uvariamicin I	501, 401, 352, 285, 217, 144, 124	AG TE
96	119.06	623.488	645.4681	2.88	$C_{37}H_{66}O_7^+$	Squamotacin	501, 352, 285, 217, 144, 124	AG TE
97	119.47		613.4808	-0.57	$C_{37}H_{67}O_5^+$	Cis-Bullatencin	493, 401, 363, 325, 303, 285, 257, 232, 176, 144	AnsTE
98	119.47		645.4705	-0.98	$C_{37}H_{67}O_7^+$	Squamocin G	613, 591, 493, 428, 401, 363, 352, 320, 303, 285, 257, 2323, 212, 142	AnsTE
99	119.86		615.4931	7.26	$C_{37}H_{69}O_5^+$	19-Epiuvariamicin III	601, 544, 401, 352, 327, 284	AG TE
100	120.58		643.4592	-7.97	$C_{37}H_{65}O_7^+$	Mosinone A	601, 579, 501, 401, 384, 352, 285, 217, 184	AnsTE
101	120.58		615.4967	-0.92	$C_{37}H_{69}O_5^+$	Reticulatain 2	579, 501, 401, 352, 285, 217, 184, 144, 124, 90	AnsTE
102	120.58		601.5411	-0.94	$C_{37}H_{61}O_6^+$	Squamocin I	501, 391, 285, 144	AnsTE
103	120.88	593.513	615.4965	-0.78	$C_{37}H_{69}O_5^+$	Squafosacin G	565, 501, 401, 352, 285, 217, 144, 124, 64	AnsTE
104	120.88		615.4965	-0.78	$C_{37}H_{69}O_5^+$	Reticulatain 1	601, 501, 401, 352, 285, 217, 144, 124, 103, 64	AnsTE, AG TE
105	120.98		645.4678	2.99	$C_{37}H_{67}O_7^+$	Squamocin F	601, 501, 401, 352, 285, 144, 124	AS, AG TE
106	122.49		629.4723	2.36	$C_{37}H_{67}O_6^+$	Squamocin M	501, 401, 352, 310, 285, 144, 124, 103	AnsTE, AG TE

107	125.02		615.491	7.89	$C_{37}H_{69}O_5^+$	15-Epiuvariamicin I	501, 436, 413, 391, 352, 338, 285, 144, 124, 103	AG TE
108	125.32		615.4933	4.99	$C_{37}H_{69}O_5^+$	17-Epiuvariamicin II	593, 501, 391, 352, 338, 285, 144, 124	AG TE
109	125.93		629.4754	-0.44	$C_{37}H_{67}O_6^+$	Squamotin C	565, 501, 401, 338, 285, 217, 144	AnsTE
110	125.93	607.494	629.4763	-1.22	$C_{37}H_{67}O_6^+$	Neoannonin B	501, 419, 338, 217, 144, 124, 64	AnsTE
111	126.03		629.4755	-0.2	$C_{37}H_{67}O_6^+$	Squamocin N	501, 338, 285, 144, 124, 64	AnsTE
112	126.03		629.4723	2.34	$C_{37}H_{67}O_6^+$	Squamocin L	338, 312, 144, 103	AnsTE, AG TE
113	126.94	607.495	629.4752	0	$C_{37}H_{67}O_6^+$	Squamotin B	565, 501, 401, 338, 285, 217, 144	AnsTE
114	127.14		629.4759	-0.6	$C_{37}H_{67}O_6^+$	Squamocin J	501, 401, 338, 285, 144	AnsTE

Table S2: Results of the minimum inhibitory concentration (MIC) of the extracts

	AngTE	Ang-AgNPs mg/ml	AnsTE	Ans-AgNPs
Staphylococcus aureus	8.25	0.75	8.25	0.375
Candida albicans	8.25	0.375	4.125	0.093
Pseudomonas aeuroginosa	16.5	0.187	16.5	0.75
Escherichia coli	16.5	1.5	16.5	0.375

Table S3. The IC_{50} (µg) of the ethanolic extracts of *A. squamosa, A. glabra,* and their nanoparticles against different human solid tumor cells.

Sample	IC_{50} (µg)		
	PC3	SKOV 3	HeLa
AngTE	6.8 ± 0.8	9.1 ± 0.6	5.7 ± 0.3
Ang-AgNPs	2.4 ± 0.3	2.8 ± 0.23	1.6 ± 0.1
AnsTE	3.5 ± 0.1	1.6 ± 0.1	0.001 ± 0.0001
Ans-AgNPs	1.7 ± 0.4	1.2 ± 0.1	0.001 ± 0.00014

Table S4. Molecular docking results of major identified compounds from Ang TE and Ans TE

Compound	Binding Score	Interacting Group (Nearest amino acid residues)	Hydrogen bond	Bond length	PDB ID
**Navitoclax (ABT-263)**	-10.76	PHE101, TYR105, ASP108, GLY142, ARG143, ASN140 ASP108, LEU134	GLY142<br>TYR199	2.24<br>2.34	4LVT
**Cis-Annonacin**	-10.59	PHE101, TYR105, ASP108, ASN140, GLY142, ARG143	ASN140<br>ARG143	2.08<br>2.27	4LVT
**Squamocin G**	-10.68	PHE101, TYR105, ARG104, ASN140, GLY142, ARG143	ASP137<br>ASN140<br>ARG143	2.15<br>2.20	4LVT
**Motrilin**	-10.74	PHE101, TYR105, ASP108, GLU133, GLY142, LEU198	ASP137<br>ASN140<br>ARG143	2.91<br>2.02	4LVT
**Squamocin**	-10.56	PHE101, TYR105, ARG138, ASN140, GLY142, ARG143	ARG104<br>ASN140<br>ARG143	2.22<br>2.04	4LVT
**Mosinone A**	-10.87	PHE101, TYR105, ASP108, VAL130, ASN140, GLY142	ARG104<br>ASN140<br>ARG143	2.33<br>2.15	4LVT
**Squamocin L**	-9.78	PHE101, TYR105, ASP108, MET112, ARG126, GLY142,	ASN140<br>ARG143	2.14	4LVT
**Bullatacinone**	-9.91	ASP100, PHE101, TYR105, ASP137, GLY142, TYR199	ARG104<br>ARG143	2.31<br>2.18	4LVT
**Squamone**	-8.49	PHE101, TYR105, ASP108, GLY142, PHE195, LEU198	ASN140<br>ARG143	2.91	4LVT
**Navitoclax analogue**	-9.80	PHE101, TYR105, GLU133, GLY142, ARG143 TYR199	GLY142<br>ASP100	3.26<br>2.79	4MAN
**Cis-Annonacin**	-9.92	ASP100, ASP137, ASN140, GLY142, ARG143, TYR199	ARG104<br>GLY142<br>ARG143	2.05-2.61<br>2.17<br>2.40	4MAN
**Squamocin G**	-9.73	ASP100, ARG104, TYR105, ASN 140, GLY142, TYR199	ASP100<br>ARG104<br>ASP137	3.00<br>2.37<br>…..	4MAN
**Motrilin**	-9.69	GLN92, ASP100, TYR105, ASN140, GLY142, LEU198	ASP100<br>ARG104<br>TRP141	2.95<br>2.45<br>2.07	4MAN
**Squamocin**	-9.76	GLN92, ASP100, TYR105, LEU134, GLY142, TYR199	ASN140<br>ARG143	2.50<br>2.35-2.38	4MAN
**Mosinone A**	-9.48	GLN92, ASP100, ASP108, LEU134, ASN140, TYR199	TYR105<br>ARG143	2.06<br>2.02	4MAN
**Squamocin L**	-8.16	ALA97, ASP100, TYR105, ASN140, PRO201	GLY142	2.34	4MAN
**Bullatacinone**	-8.96	ALA97, ASP100, TYR105 ASN140, GLY142, ARG143	ASN140<br>ARG143	2.09<br>2.45	4MAN

**Squamone**	-7.76	ALA97, ASP100, TYR105 ASP108, ASN140, GLY142	ARG143	2.37	4MAN
